# Raincloud plots: a multi-platform tool for robust data visualization

**DOI:** 10.12688/wellcomeopenres.15191.2

**Published:** 2021-01-21

**Authors:** Micah Allen, Davide Poggiali, Kirstie Whitaker, Tom Rhys Marshall, Jordy van Langen, Rogier A. Kievit

**Affiliations:** 1 Aarhus Institute of Advanced Studies, Aarhus University, Aarhus, Denmark; 2 Department of Psychiatry, University of Cambridge, Cambridge, UK; 3 Centre of Functionally Integrative Neuroscience, Aarhus University Hospital, Aarhus, Denmark; 4 Department of Mathematics, University of Padova, Padova, Italy; 5 Padova Neuroscience Center, University of Padova, Padova, Italy; 6 Alan Turing Institute, London, UK; 7 Wellcome Centre for Integrative Neuroimaging, University of Oxford, Oxford, UK; 8 Department of Experimental Psychology, University of Oxford, Oxford, UK; 9 Donders Institute for Brain, Cognition and Behavior, Radboud University, Nijmegen, New Zealand; 10 Max-Planck Centre for Computational Psychiatry and Aging, UCL/MPI Berlin, London, UK; 11 MRC Cognition and Brain Sciences Unit, University of Cambridge, Cambridge, UK

**Keywords:** data visualization, raincloud plots, R, Python, Matlab, barplots

## Abstract

Across scientific disciplines, there is a rapidly growing recognition of the need for more statistically robust, transparent approaches to data visualization. Complementary to this, many scientists have called for plotting tools that accurately and transparently convey key aspects of statistical effects and raw data with minimal distortion. Previously common approaches, such as plotting conditional mean or median barplots together with error-bars have been criticized for distorting effect size, hiding underlying patterns in the raw data, and obscuring the assumptions upon which the most commonly used statistical tests are based. Here we describe a data visualization approach which overcomes these issues, providing maximal statistical information while preserving the desired ‘inference at a glance’ nature of barplots and other similar visualization devices. These “raincloud plots” can visualize raw data, probability density, and key summary statistics such as median, mean, and relevant confidence intervals in an appealing and flexible format with minimal redundancy. In this tutorial paper, we provide basic demonstrations of the strength of raincloud plots and similar approaches, outline potential modifications for their optimal use, and provide open-source code for their streamlined implementation in R, Python and Matlab ( https://github.com/RainCloudPlots/RainCloudPlots). Readers can investigate the R and Python tutorials interactively in the browser using Binder by Project Jupyter.

## Introduction

Effective data visualization is key to the interpretation and communication of data analysis. Ideally a statistical plot or data graphic should balance functionality, interpretability, and complexity, all without needlessly sacrificing aesthetics. That is to say, the perfect visualization is one which uses as little ‘ink’ as possible to capture exactly the desired statistical inference in an intuitive and appealing format ( Tufte, 1983). As concerns regarding the need for robust, reproducible data science have grown in recent years, so too have calls for more meaningful approaches to plotting one’s data. Here we present an open source, multi-platform tutorial for the *raincloud plot* ( Neuroconscience, 2018a).

**Figure 1.**
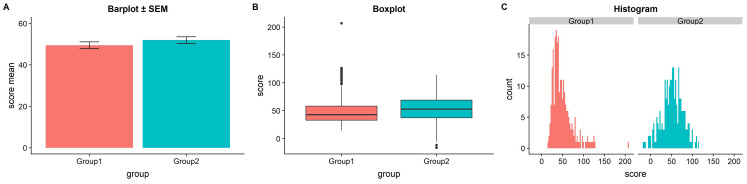
The trouble with barplots. Example reproduced from “Boxplots vs. Barplots” ( 2016) two simulated datasets with mean = 50, sd = 25, and 1000 observations. **A**) a barplot and errorbars representing +/- standard error of the mean gives the impression that the measure is equivalent between the two groups. In fact, group 1 is drawn from an exponential distribution as seen in **B**) boxplots, and **C**) histograms. The barplot not only obscures the underlying nature of the observations, but also hides the fact that these data are not appropriate for standard parametric inference. See figure1.Rmd for code to generate these figures.

A common visualization method of raw datapoints is the barplot (see Figure 1, left panel) to represent the mean or median of some condition or group via horizontal bars (or lines) and represents uncertainty about the illustrated parameter estimated via ‘whisker’ errorbars, usually conveying the standard error or 95% confidence interval. This approach has been widely criticized on several counts, including: 1) it is prone to distortion (e.g., by cropping of the Y-axis), 2) it fails to represent the actual data underlying relevant parameter inferences, 3) it often leads to misleading inferences about the magnitudes of statistical differences between conditions ( Weissgerber *et al*., 2015) and 4) it may obscure differences in distributions (and concurrent violations of distributional assumptions in parametric statistics). These limitations are illustrated in Figure 1, below. Indeed, criticism of this approach has reached such a pitched fervor that a movement to “bar bar plots” ( “#barbarplots,” 2016; Piccinini, 2016) has arisen with many signees pledging to request all such plots be changed to something more informative ^1^.

**Figure 2.**
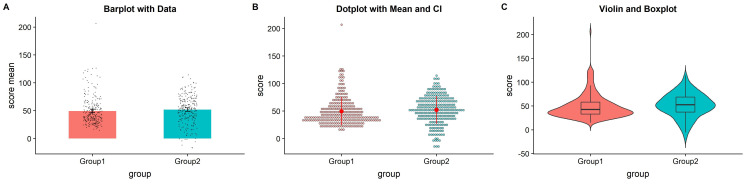
Extant approaches to improved data plotting. **A**) The simplest improvement is to add jittered raw data points to the standard boxplot and +/- standard error scheme. **B**) Alternatively, dotplots can be used to supplement visualizations of central tendency and error, at the risk of added complexity due to the dependence of such plots on choices such as bin-width and dot size. **C**) A popular recent alternative is the violin plot coupled with boxplots or similar. However, this needlessly mirrors information about the redundant data axis (here, the x-axis). See figure2.Rmd for code to generate these figures.

To remedy these shortcomings, a variety of visualisation approaches have been proposed, illustrated in Figure 2, below. One simple improvement is to overlay individual observations (datapoints) beside the standard bar-plot format, typically with some degree of randomized jitter to improve visibility ( Figure 2A). Complementary to this approach, others have advocated for more statistically robust illustrations such as boxplots ( Tukey, 1970), which display sample median alongside interquartile range. Dot plots can be used to combine a histogram-like display of distribution with individual data observations ( Figure 2B). In many cases, particularly when parametric statistics are used, it is desirable to plot the distribution of observations. This can reveal valuable information about how e.g., some condition may increase the skewness or overall shape of a distribution. In this case, the ‘violin plot’ ( Figure 2C) which displays a probability density function of the data mirrored about the uninformative axis is often preferred ( Hintze & Nelson, 1998). With the advent of increasingly flexible and modular plotting tools such as ggplot2 ( Wickham, 2010; Wickham & Chang, 2008), all of the aforementioned techniques can be combined in a complementary fashion.

Indeed, this combined approach is typically desirable as each of these visualization techniques have various trade-offs. Simply plotting raw data can reveal valuable information about individual differences, outliers, and unexpected patterns within the data. However, human observers are notoriously poor ^2^ at estimating statistical moments and distributions from raw data ( Bobko & Karren, 1979; “Guess the Correlation,” 2017; Spence *et al*., 2016; Zylberberg *et al*., 2014), and the utility of such plots can be limited when the number of observations is large. In this case the dotplot may be advantageous, as it displays both a histogram of raw data points and the frequency of different binned observations. On the other hand, the interpretation of dotplots depends heavily on the choice of dot-bin and dot-size, and these plots can also become extremely difficult to read when there are many observations. The violin plot in which the probability density function (PDF) of observations are mirrored, combined with overlaid boxplots, have recently become a popular alternative. This provides both an assessment of the data distribution and statistical inference at a glance (SIG) via overlaid boxplots ^3^. However, there is arguably little to be gained, statistically speaking, by mirroring the PDF in the violin plot, and therefore they are violating the philosophy of minimising the “data-ink ratio” ( Tufte, 1983) ^1^.

**Figure 3.**
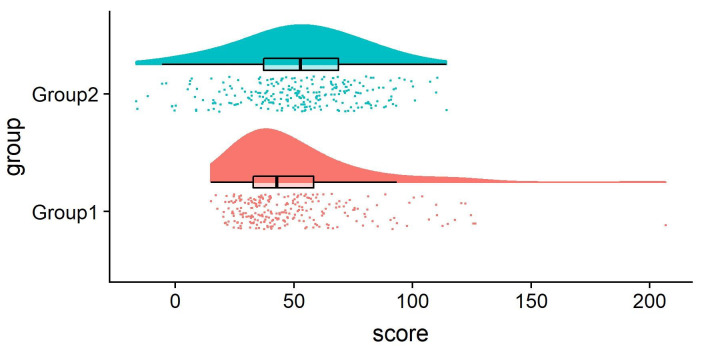
Example Raincloud plot. The raincloud plot combines an illustration of data distribution (the ‘cloud’), with jittered raw data (the ‘rain’). This can further be supplemented by adding boxplots or other standard measures of central tendency and error. See figure3.Rmd for code to generate this figure.

To overcome these issues, we propose the use of the ‘raincloud plot’ ( Neuroconscience, 2018a), illustrated in Figure 3. The raincloud plot combines a wide range of visualization suggestions, and similar precursors have been used in various publications (e.g., Ellison, 1993, Figure 2.4; Wilson *et al*., 2018). The plot attempts to address the aforementioned limitations in an intuitive, modular, and statistically robust format. In essence, raincloud plots combine a ‘split-half violin’ (an un-mirrored PDF plotted against the redundant data axis), raw jittered data points, and a standard visualization of central tendency (i.e., mean or median) and error, such as a boxplot. As such the raincloud plot builds on code elements from multiple developers and scientific programming languages ( Hintze & Nelson, 1998; Patil, 2018; Wickham & Chang, 2008; Wilke, 2017).

Many previous attempts have been made to produce more robust, intuitive, and transparent plots. Our goal here is not to propose a totally novel invention, but rather to make a powerful visualization strategy freely, easily, and transparently available across commonly used platforms. To this end, similar but distinct plotting strategies include beanplots ( Kampstra, 2008), estimation plots ( Ho *et al*., 2018), pirateplots ( Phillips, 2016), sinaplots ( Sidiropoulos *et al*., 2018), stripcharts ( Chambers, 2017), beeswarm plots ( Eklund, 2016), and many others. Moreover, there are likely settings where rainclouds may not necessarily be ideal, such as when there is an extreme number of (repeated measures) datapoints, or a large number of waves, that render the points or density plots confusing rather than illuminating. Conversely, there are settings such as simple counts, proportions, and frequencies when oft-dreaded barplots may be adequate tools. No data visualization tool will be ideal for all settings, but we think raincloudplots are a new, flexible tool that could be considered in many common scenarios. Our hope here is to offer a cross-platform, open science tool which builds upon these approaches and makes robust and transparent data-plotting available to as wide an audience as possible.

Inference-at-a-glance is supported by adding whatever flavor of data summary measure is optimal for the data at hand; typical examples include overlaid boxplots or other illustrations of central tendency such as mean/median and associated confidence intervals. Depending on the analysis at hand, PDF illustration can also be replaced with more advanced options such as posterior probability densities (i.e., as derived from Bayesian inference) or other parameter estimates ( Ho *et al*., 2018).

**Figure 4.**
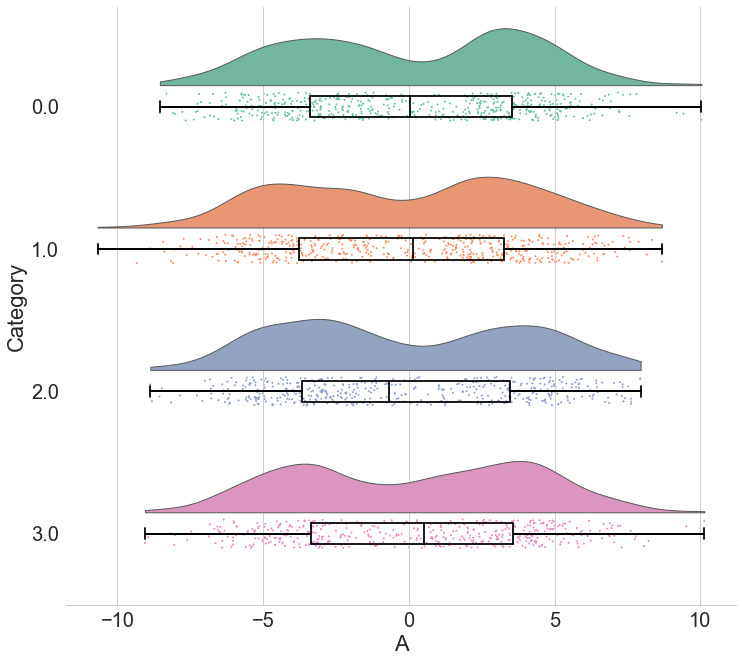
Raincloud plots leave little to the imagination. By replacing the redundantly mirrored probability distribution with a boxplot and raw data-points, the raincloud plot provides the user with information both about individual observations and patterns among them (such as striation or clustering), and overall tendencies in the distribution. As illustrated here, even a boxplot plus raw data may hide bimodality or other crucial facets of the data. See figure4.ipynb for code to generate these figures.

Thus, raincloud plots offer the user maximum utility and flexibility, ensuring that nothing is ‘hidden away’ and that the reader has all information needed to assess the data, its distribution, and the appropriateness of any reported statistical tests in a visually appealing format. Indeed, as illustrated in Figure 4, raincloud plots can reveal information that even a boxplot plus raw data might hide away, such as a bimodal distribution which may not be readily ‘eyeballed’ from raw data points.

In terms of general interest, following their introduction raincloud plots have generated substantial enthusiasm on social media amongst scientists from a variety of disciplines ( @neuroconscience, 2018b; Neuroconscience, 2018a), and are now available as a default option in at least one statistical plotting software ( Wilke, 2017). To further their accessibility and ease-of-use, in the following multi-platform tutorial we provide code and documentation for the step-by-step creation and customization of raincloud plots in R, Matlab, and Python.

## Code tutorials: how to make it rain

### How to make it rain in R

R ( https://www.r-project.org) is a multiplatform, free and open source tool widely used in the statistical community ( R Core Team, 2013). Our tutorial includes an associated R-script to create the raincloud function which complements the existing ggplot2 package ( Wickham, 2010; Wickham & Chang, 2008), as well as an R-notebook (reproduced below) which walks the user through the simulation of data, illustrates a variety of parameters that can be user modified and shows how to get from barplots to rainclouds.

There are two ways to create raincloudplots in R: Through a series of specific easy to modify scripts, and through our new tailored package, ‘raincloudplots’. The former provides a step-by-step walkthrough with individual scripts which can be modified as needed. The latter provides an easy-to-use set of functions for the most common experimental designs and data formats. The full package tutorial is available here: https://github.com/jorvlan/raincloudplots

First, we demonstrate the individual script tutorial. The code is available at https://github.com/RainCloudPlots/RainCloudPlots/tree/master/tutorial_R and can be run interactively in the browser at

https://mybinder.org/v2/gh/RainCloudPlots/RainCloudPlots/master?urlpath=rstudio.

This tutorial will walk you through the process of transforming your barplots into rainclouds, and also show you how to customize your rainclouds for various options such as ordinal or repeated measures data. First, we’ll run the included “R_rainclouds” script, which will set-up the split-half violin option in ggplot, as well as simulate some data for our figures:



source("R_rainclouds.R")
source("summarySE.R")
source("simulateData.R")
library(cowplot)
library(readr)
# width and height variables for saved plots
w = 6
h = 3

head(summary_simdat)
##    group   N score_mean score_median       sd       se       ci
## 1 Group1 250   49.45877     42.74587 25.27975 1.598832 3.148958
## 2 Group2 250   51.94353     52.69956 25.06328 1.585141 3.121994



The function gives us two groups of N = 250 observations each; both have similar means and SDs, but group one is drawn from an exponential distribution. Now we’ll plot a basic barplot for our simulated data. Note that we’re using the ‘cowplot’ theme ( https://github.com/wilkelab/cowplot) to produce simple, uncluttered plots - you should set-up your own theme or other customization options as desired:


#Barplot
p1 <- ggplot(summary_simdat, aes(x = group, y = score_mean, fill = group))+
  geom_bar(stat = "identity", width = .8)+
  geom_errorbar(aes(ymin = score_mean - se, ymax = score_mean+se), width = .2)+
  guides(fill=FALSE)+
  ylim(0, 80)+
  ylab('Score')+xlab('Group')+theme_cowplot()+
  ggtitle("Figure R1: Barplot +/- SEM")
  ggsave('1Barplot.png', width = w, height = h)
  
p1 



**Figure:**
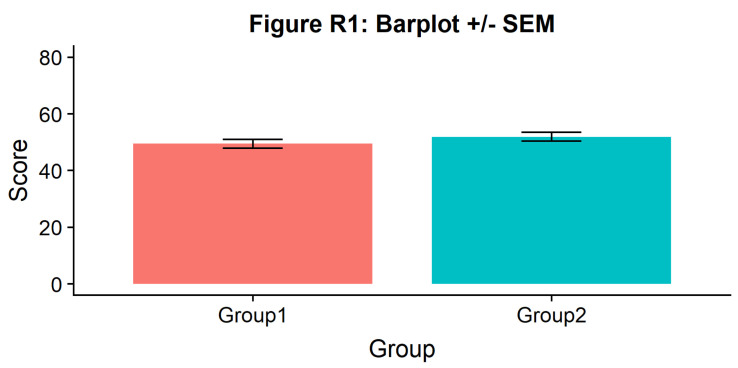


There we go - just needs some little asterisks and we’re ready to publish! Just kidding. Let’s start our first, most basic raincloud plot like so, using the ‘geom_flat_violin’ option our function already setup for us:


#Basic plot
p2 <- ggplot(simdat,aes(x=group,y=score))+
  geom_flat_violin(position = position_nudge(x = .2, y = 0),adjust =2)+
  geom_point(position = position_jitter(width = .15), size = .25)+
  ylab('Score')+xlab('Group')+theme_cowplot()+
  ggtitle('Figure R2: Basic Rainclouds or Little Prince Plot')+
  ggsave('2basic.png', width = w, height = h)

p2



**Figure:**
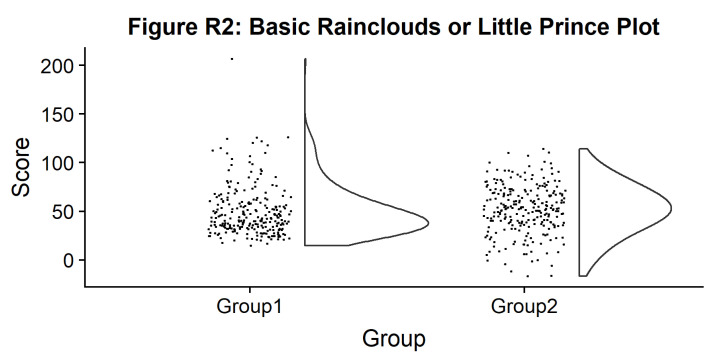


Now we can see the raw data (our ‘rain’), and the overlaid probability distribution (the ‘cloud’). Let’s make it a bit prettier and easier to read by adding some colours. We can also use ‘coordinate flip’ to rotate the entire plot about the x-axis, transforming our ‘little prince plots’ into true rainclouds:


#Plot with colours and coordinate flip
p3 <- ggplot(simdat,aes(x=group,y=score, fill = group))+
  geom_flat_violin(position = position_nudge(x = .2, y = 0),adjust = 2)+
  geom_point(position = position_jitter(width = .15), size = .25)+
  
ylab('Score')+xlab('Group')+coord_flip()+theme_cowplot()+guides(fill = FALSE)+
  ggtitle('Figure R3: The Basic Raincloud with Colour')+
  ggsave('figs/rTutorial/3pretty.png', width = w, height = h)

p3


**Figure:**
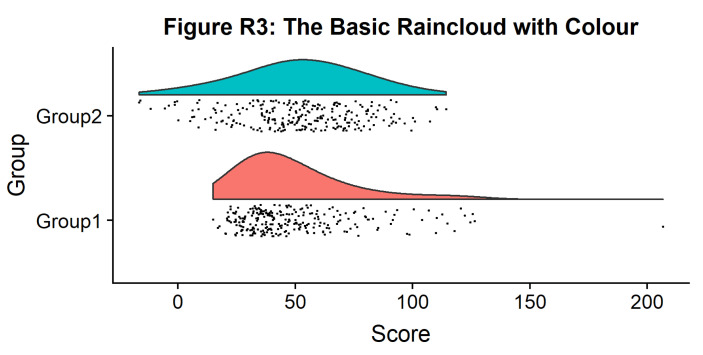


In case you want to change the smoothing kernel used to calculate the PDFs, you can do so by altering the ‘adjust’ flag for geom_flat_violin. For example, here we’ve dropped our smoothing to give a much bumpier raincloud:


#Raincloud with reduced smoothing
p4 <- ggplot(simdat,aes(x=group,y=score, fill = group))+
  geom_flat_violin(position = position_nudge(x = .2, y = 0),adjust = .2)+
  geom_point(position = position_jitter(width = .15), size = .25)+
  
ylab('Score')+xlab('Group')+coord_flip()+theme_cowplot()+guides(fill = FALSE) + 
  ggtitle('Figure R4: Unsmooth Rainclouds')
  ggsave('4unsmooth.png', width = w, height = h)

p4


**Figure:**
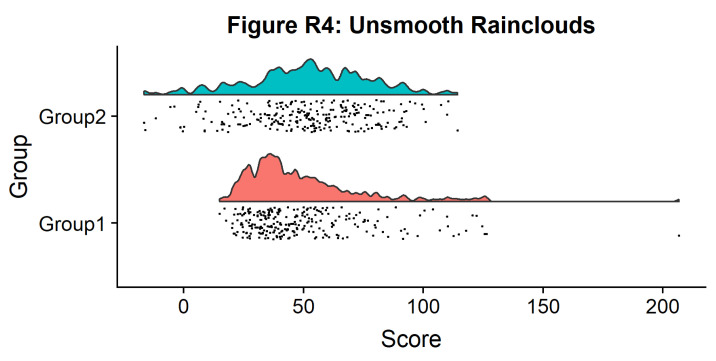


Now we need to add something to help us easily evaluate any possible differences between our groups or conditions. To achieve this, we’ll add some boxplots to complete our raincloud plots. To get the boxplots to line up however we like, we need to set our x-axis to a numeric value, so we can add a fixed offset:


#Rainclouds with boxplots
p5 <- ggplot(simdat,aes(x=group,y=score, fill = group))+
  geom_flat_violin(position = position_nudge(x = .25, y = 0),adjust =2)+
  geom_point(position = position_jitter(width = .15), size = .25)+
#note that here we need to set the x-variable to a numeric variable and bump it to get the boxplots to line up with the rainclouds. 
  geom_boxplot(aes(x = as.numeric(group)+0.25, y = score),outlier.shape = NA, alpha = 0.3, width = .1, colour = "BLACK") + 
  
ylab('Score')+xlab('Group')+coord_flip()+theme_cowplot()+guides(fill = FALSE, colour = FALSE) + 
  ggtitle("Figure R5: Raincloud Plot w/Boxplots")
  ggsave('5boxplots.png', width = w, height = h)

p5


**Figure:**
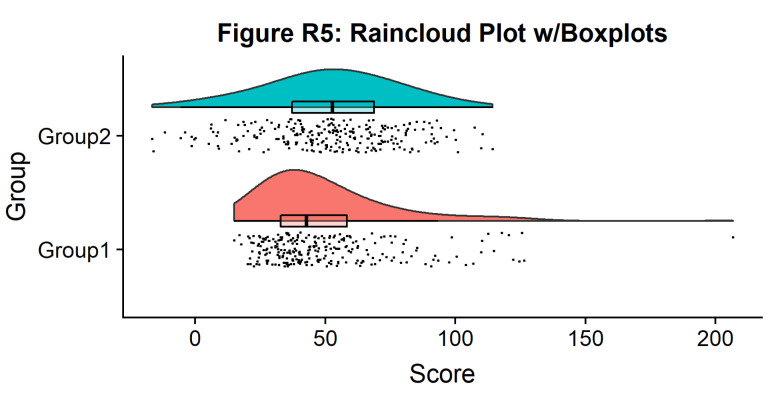


Now we’ll make a few aesthetic tweaks. You may want to turn these on or off depending on your preferences. We’ll take the black outline away from the plots by adding the colour = group parameter, and we’ll also change colour palettes using the built-in colour brewer tool.


#Rainclouds with boxplots
p6 <- ggplot(simdat,aes(x=group,y=score, fill = group, colour = group))+
  geom_flat_violin(position = position_nudge(x = .25, y = 0),adjust =2, trim = FALSE)+
  geom_point(position = position_jitter(width = .15), size = .25)+
  geom_boxplot(aes(x = as.numeric(group)+0.25, y = score),outlier.shape = NA, alpha = 0.3, width = .1, colour = "BLACK") +
  
ylab('Score')+xlab('Group')+coord_flip()+theme_cowplot()+guides(fill = FALSE, colour = FALSE) +
  scale_colour_brewer(palette = "Dark2")+
  scale_fill_brewer(palette = "Dark2")+
  ggtitle("Figure R6: Change in Colour Palette")
  ggsave('6boxplots.png', width = w, height = h)
  
p6



**Figure:**
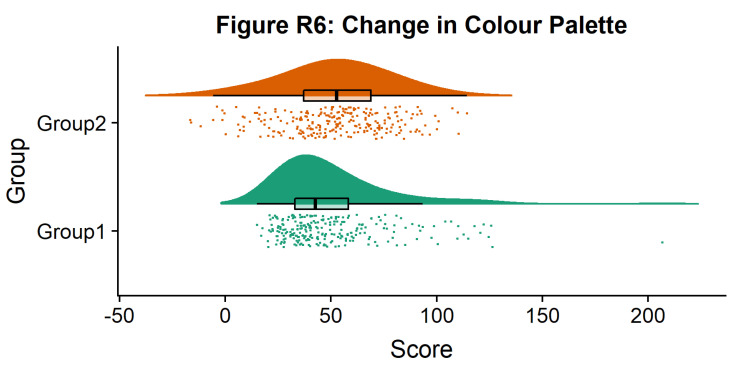


Alternatively, you may prefer to simply plot mean or median with standard confidence intervals. Here we’ll plot the mean as well as 95% confidence intervals, which we’ve calculated using the included SummarySE function (from https://www.rdocumentation.org/packages/Rmisc/versions/1.5/topics/summarySE), by overlaying them on of our clouds:


#Rainclouds with mean and confidence interval
p7 <- ggplot(simdat,aes(x=group,y=score, fill = group, colour = group))+
  geom_flat_violin(position = position_nudge(x = .25, y = 0),adjust =2)+
  geom_point(position = position_jitter(width = .15), size = .25)+
  geom_point(data = summary_simdat, aes(x = group, y = score_mean), position = position_nudge(.25), colour = "BLACK")+
  geom_errorbar(data = summary_simdat, aes(x = group, y = score_mean, ymin = score_mean-ci, ymax = score_mean+ci), position = position_nudge(.25), colour = "BLACK", width = 0.1, size = 0.8)+
  
ylab('Score')+xlab('Group')+coord_flip()+theme_cowplot()+guides(fill = FALSE, colour = FALSE) +
  scale_colour_brewer(palette = "Dark2")+
  scale_fill_brewer(palette = "Dark2")+
  ggtitle("Figure R7: Raincloud Plot with Mean Â± 95% CI")
  ggsave('7meanplot.png', width = w, height = h)

p7



**Figure:**
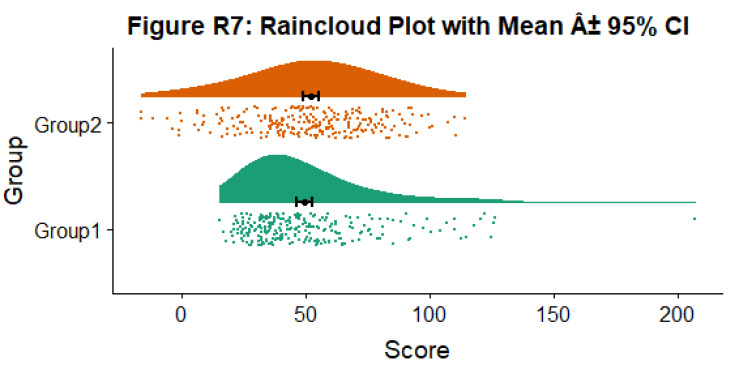


If your data is discrete or ordinal you may need to manually add some jitter to improve the plot:


#Rainclouds with striated data

#Round data
simdat_round<-simdat
simdat_round$score<-round(simdat$score,0) 

#Striated/grouped when no jitter applied
ap1 <- ggplot(simdat_round,aes(x=group,y=score,fill=group,col=group))+geom_flat_violin(position = position_nudge(x = .2, y = 0), alpha = .6,adjust =4)+geom_point(size = 1, alpha = 0.6)+ylab('Score')+scale_fill_brewer(palette = "Dark2")+scale_colour_brewer(palette = "Dark2")+guides(fill = FALSE, col = FALSE)+ggtitle('Striated')




#Added jitter helps
ap2 <-
ggplot(simdat_round,aes(x=group,y=score,fill=group,col=group))+geom_
flat_violin(position = position_nudge(x = .2, y = 0), alpha =
.4,adjust =4)+geom_point(position=position_jitter(width = .15),size 
= 1, alpha = 0.4)+ylab('Score')+scale_fill_brewer(palette =
"Dark2")+scale_colour_brewer(palette = "Dark2")+guides(fill = FALSE,
col = FALSE)+ggtitle('Added jitter')



all_plot <- plot_grid(ap1, ap2, labels="AUTO")



# add title to cowplot
title <- ggdraw() + 
  draw_label("Figure R8: Jittering Ordinal Data",
              fontface = 'bold')



all_plot_final <- plot_grid(title, all_plot, ncol = 1, rel_heights =
c(0.1, 1)) # rel_heights values control title margins



ggsave('8allplot.png', width = w, height = h)
all_plot_final


**Figure:**
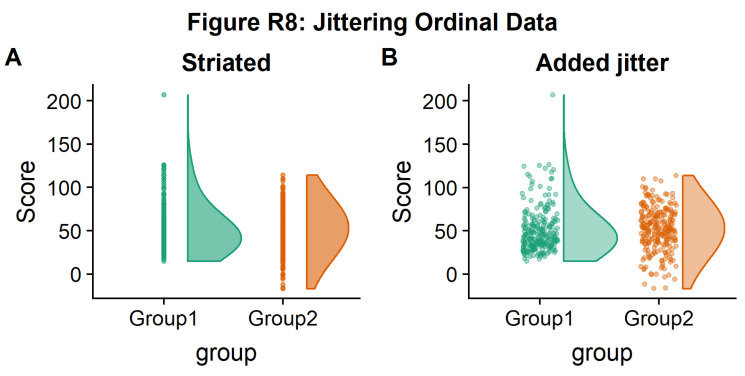


Finally, in many situations you may have nested, factorial, or repeated measures data. In this case, one option is to use plot facets to group by factor, emphasizing pairwise differences between conditions or factor levels:


#Add additional factor/condition
simdat$gr2<-as.factor(c(rep('high',125),rep('low',125),rep('high',125),rep('low',125)))



p9 <- ggplot(simdat,aes(x=group,y=score, fill = group, colour = group))+
  geom_flat_violin(position = position_nudge(x = .25, y = 0),adjust =2, trim = TRUE)+
  geom_point(position = position_jitter(width = .15), size = .25)+
  geom_boxplot(aes(x = as.numeric(group)+0.25, y = score),outlier.shape = NA, alpha = 0.3, width = .1, colour = "BLACK") +
  
  
ylab('Score')+xlab('Group')+coord_flip()+theme_cowplot()+guides(fill = FALSE, colour = FALSE) + facet_wrap(~gr2)+
  scale_colour_brewer(palette = "Dark2")+
  scale_fill_brewer(palette = "Dark2")+
  ggtitle("Figure R9: Complex Raincloud Plots with Facet Wrap")
  ggsave('9facetplot.png', width = w, height = h)

p9



**Figure:**
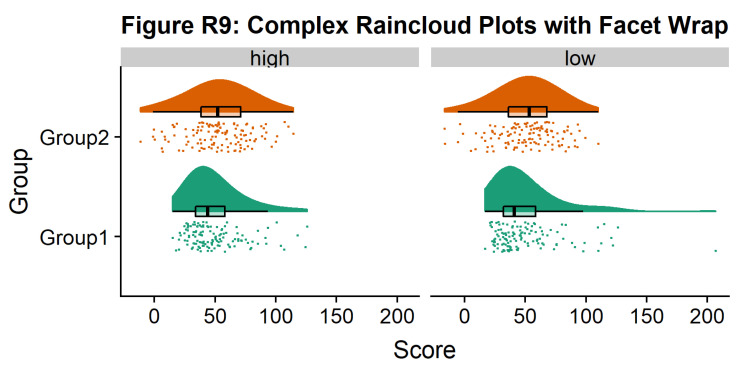


As another example, we consider some simulated repeated measures data in factorial design, where two groups are measured across three timepoints. To do so, we’ll first load in some new data:


#load the repeated measures factorial data

rep_data <- read_csv("data/repeated_measures_data.csv", 
    col_types = cols(group = col_factor(levels = c("1", 
        "2")), time = col_factor(levels = c("1", 
        "2", "3"))))

sumrepdat <- summarySE(rep_data, measurevar = "score",
groupvars=c("group", "time"))




head(sumrepdat)
##   group time  N score_mean score_median       sd        se        ci
## 1     1    1 18   6.362222        6.670 1.658861 0.3909972 0.8249319
## 2     1    2 18   7.468333        7.730 1.546880 0.3646032 0.7692454
## 3     1    3 18  10.482778       10.455 1.060254 0.2499043 0.5272520
## 4     2    1 11   1.847273        1.210 2.010279 0.6061219 1.3505238
## 5     2    2 11   3.684545        2.920 2.135108 0.6437594 1.4343852
## 6     2    3 11   7.358182        7.020 2.236273 0.6742616 1.5023486


Now, we’ll plot our rainclouds with boxplots again, this time adding some dodge so we can better emphasize differences between our factors and factor levels. Note that here we need to nudge the point x-axis as a numeric valuable, as this work around does not currently work for boxplots with multiple factors:


# Rainclouds for repeated measures, continued 
p10 <- ggplot(rep_data, aes(x = time, y = score, fill = group)) +
  geom_flat_violin(aes(fill = group),position = position_nudge(x = .1, y = 0), adjust = 1.5, trim = FALSE, alpha = .5, colour = NA)+
  geom_point(aes(x = as.numeric(time)-.15, y = score, colour = group),position = position_jitter(width = .05), size = 1, shape = 20)+
  geom_boxplot(aes(x = time, y = score, fill = group),outlier.shape = NA, alpha = .5, width = .1, colour = "black")+
  scale_colour_brewer(palette = "Dark2")+
  scale_fill_brewer(palette = "Dark2")+
  ggtitle("Figure R10: Repeated Measures Factorial Rainclouds")
  ggsave('10repanvplot.png', width = w, height = h)
  #coord_flip()+
p10



**Figure:**
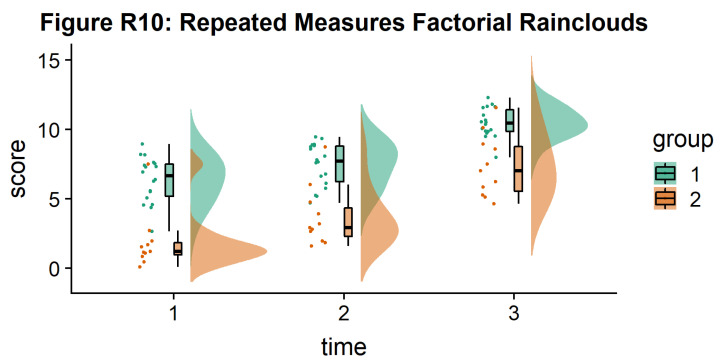


Finally, you may want to add traditional line plots to emphasize factorial interactions and main effects. Here we’ve plotted the mean and standard error for each cell of our design and connected these with a hashed line. There are a lot of possible options though, so you’ll need to decide what works best for your needs:


#Rainclouds for repeated measures, additional plotting options 

p11 <- ggplot(rep_data, aes(x = time, y = score, fill = group)) +
  geom_flat_violin(aes(fill = group),position = position_nudge(x = .1, y = 0), adjust = 1.5, trim = FALSE, alpha = .5, colour = NA)+
  geom_point(aes(x = as.numeric(time)-.15, y = score, colour = group),position = position_jitter(width = .05), size = .25, shape = 20)+
  geom_boxplot(aes(x = time, y = score, fill = group),outlier.shape = NA, alpha = .5, width = .1, colour = "black")+
  geom_line(data = sumrepdat, aes(x = as.numeric(time)+.1, y = score_mean, group = group, colour = group), linetype = 3)+
  geom_point(data = sumrepdat, aes(x = as.numeric(time)+.1, y = score_mean, group = group, colour = group), shape = 18) +
  geom_errorbar(data = sumrepdat, aes(x = as.numeric(time)+.1, y = score_mean, group = group, colour = group, ymin = score_mean-se, ymax = score_mean+se), width = .05)+
  scale_colour_brewer(palette = "Dark2")+
  scale_fill_brewer(palette = "Dark2")+
  ggtitle("Figure R11: Repeated Measures - Factorial (Extended)")
  ggsave('11repanvplot2.png', width = w, height = h)
  #coord_flip()+
  
p11



**Figure:**
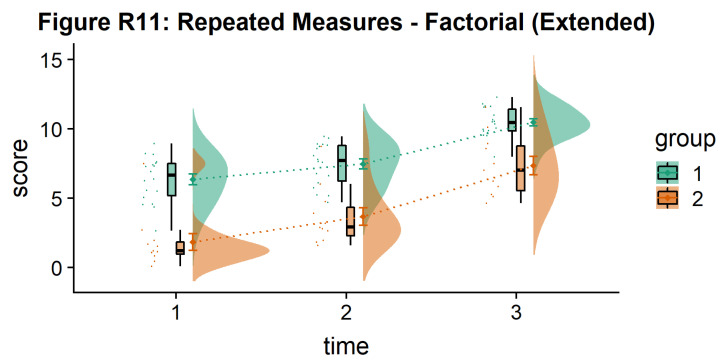


Here is the same plot, but with the grouping variable flipped:


#Rainclouds for repeated measures, additional plotting options

p12 <- ggplot(rep_data, aes(x = group, y = score, fill = time)) +
  geom_flat_violin(aes(fill = time),position = position_nudge(x = .1, y = 0), adjust = 1.5, trim = FALSE, alpha = .5, colour = NA)+
  geom_point(aes(x = as.numeric(group)-.15, y = score, colour = time),position = position_jitter(width = .05), size = .25, shape = 20)+
  geom_boxplot(aes(x = group, y = score, fill = time),outlier.shape = NA, alpha = .5, width = .1, colour = "black")+
  geom_line(data = sumrepdat, aes(x = as.numeric(group)+.1, y = score_mean, group = time, colour = time), linetype = 3)+
  geom_point(data = sumrepdat, aes(x = as.numeric(group)+.1, y = score_mean, group = time, colour = time), shape = 18) +
  geom_errorbar(data = sumrepdat, aes(x = as.numeric(group)+.1, y = score_mean, group = time, colour = time, ymin = score_mean-se, ymax = score_mean+se), width = .05)+
  scale_colour_brewer(palette = "Dark2")+
  scale_fill_brewer(palette = "Dark2")+
  ggtitle("Figure R12: Repeated Measures - Factorial (Extended)") +
  coord_flip()
  ggsave('12repanvplot3.png', width = w, height = h)

p12


**Figure:**
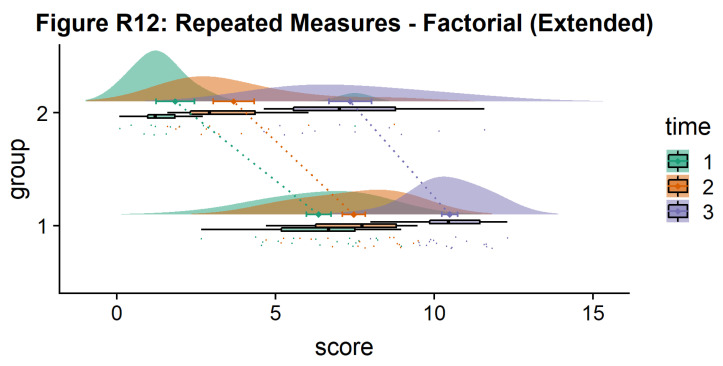


### R package - raincloudplots

In addition to this step-by-step tutorial, we have developed two other tools to visualize data in rainclouds, which are primarily suited for repeated measures data. First, we wrote an extensive GitHub tutorial called ‘open-visualizations’ ( https://github.com/jorvlan/open-visualizations) which provides detailed and extensive R code to create robust and transparent repeated measures visualizations, by showing the slope change for each individual data point over time. To date, this tutorial has been cited in 15 scientific papers. However, using this tutorial requires sufficient R programming knowledge and might therefore not be suitable for non-R experts. Therefore, we have created a dedicated raincloudplots package ( https://github.com/jorvlan/raincloudplots) written in R. This package is tailored towards easy visualization of grouped and repeated measures data. Moreover, it also provides individually linked repeated measures visualizations, which add detail and richness to a multitude of within-subject designs. Here, we have chosen to depict the two most common repeated measures designs: 1 * 1 and 2 * 2. The following examples show you some ways to use the package for simple between and within subject designs. The package contains more raincloudplots that you can make, please visit the ( https://github.com/jorvlan/raincloudplots) to see all the examples.

Install the package


if (!require(remotes)) {
    install.packages("remotes")
}
remotes::install_github('jorvlan/raincloudplots')
library(raincloudplots)

w_package = 3
h_package = 6


### 1 by 1 repeated measures

Step 1: Initialize the data-format


df_1×1 >- data_1×1(
  array_1 = iris$Sepal.Length[1:50],
  array_2 = iris$Sepal.Length[51:100],
  jit_distance = .09,
  jit_seed = 321)


Step 2: Create the plot


raincloud_1 <- raincloud_1×1_repmes(
  data = df_1×1,
  colors = (c('dodgerblue', 'darkorange')),
  fills = (c('dodgerblue', 'darkorange')),
  line_color = 'gray',
  line_alpha = .3,
  size = 1,
  alpha = .6,
  align_clouds = FALSE) +

scale_x_continuous(breaks=c(1,2), labels=c("Pre", "Post"), limits=c(0, 3)) +
  xlab("Time") +
  ylab("Score") +
  theme_classic()
ggsave('../figs/tutorial_R/package1.png', width = w_package, height = h_package)

raincloud_1


**Figure:**
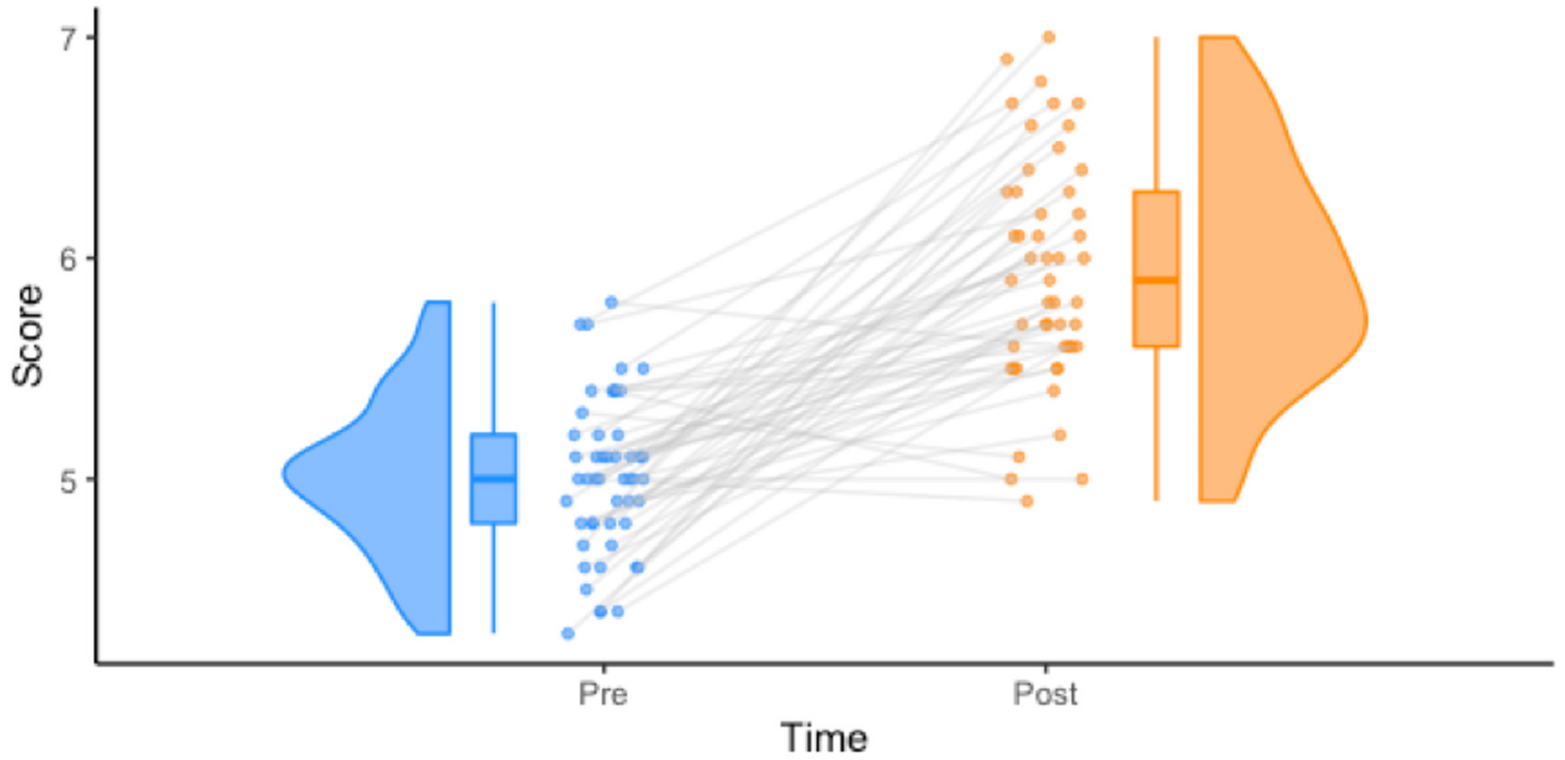


### 2 by 2 repeated measures

Step 1: Initialize the data-format


df_2×2 <- data_2×2(
  array_1 = iris$Sepal.Length[1:50],
  array_2 = iris$Sepal.Length[51:100],
  array_3 = iris$Sepal.Length[101:150],
  array_4 = iris$Sepal.Length[81:130],
  labels = (c('congruent','incongruent')),
  jit_distance = .09,
  jit_seed = 321,
  spread_x_ticks = TRUE)


Step 2: Create the plot


raincloud_2 <- raincloud_2×2_repmes(
  data = df_2×2,
  colors = (c('dodgerblue', 'darkorange', 'dodgerblue', 'darkorange')),
  fills = (c('dodgerblue', 'darkorange', 'dodgerblue', 'darkorange')),
  line_color = 'gray',
  line_alpha = .3,
  size = 1,
  alpha = .6,
  spread_x_ticks = TRUE) +

scale_x_continuous(breaks=c(1,2,3,4), labels=c("Pre", "Post", "Pre", "Post"), limits=c(0, 5)) +
  xlab("Time") + 
  ylab("Score") +
  theme_classic() 
ggsave('../figs/tutorial_R/package2.png', width = w_package, height = h_package)

raincloud_2


**Figure:**
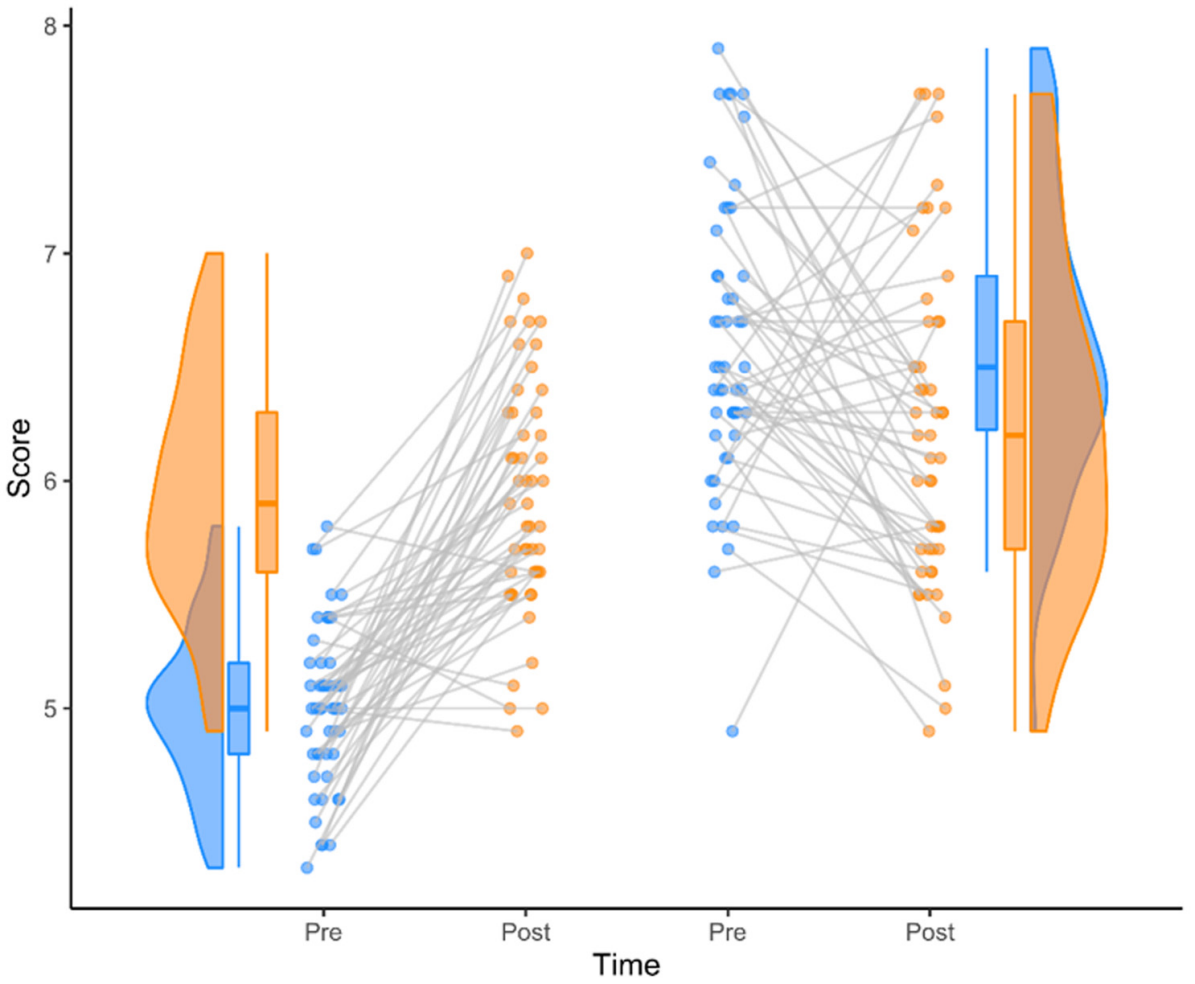


That’s it! We hope you’ll be able to use this tutorial to find great illustrations for your data, and that we’ve given you an idea of some of the different ways you can customize your raincloud plots. Next, we’ll consider how to reproduce these steps in Python and Matlab.

### How to Make it Rain in Python

Python is an open source programming language ( https://www.python.org) that has recently become extremely popular within data science and statistical machine learning. Our interactive Python tutorial can be found at the following URL:

https://github.com/RainCloudPlots/RainCloudPlots/blob/master/tutorial_python/raincloud_tutorial_python.ipynb

The tutorial follows the footsteps of the R tutorial to guide you in the creation and customization of Raincloud plots. The Python implementation of Raincloud Plots is a package named PtitPrince ( https://github.com/pog87/PtitPrince), written on the top of seaborn. Seaborn ( https://seaborn.pydata.org) is a Python plotting library written as an extension to the Python graphic library matplotlib ( https://matplotlib.org) supporting aesthetically pleasing plots and to work directly with pandas dataframes. The tutorial can be run interactively in the browser at:

https://mybinder.org/v2/gh/RainCloudPlots/RainCloudPlots/master?filepath=tutorial_python%2Fraincloud_tutorial_python.ipynb.

As first step, we will load the same dataset used before and visualize the distribution of each measure as a simple barplot with errorbars:


import pandas as pd
import ptitprince as pt
import seaborn as sns
import matplotlib.pyplot as plt
sns.set(style="whitegrid",font_scale=2)
import matplotlib.collections as clt

df = pd.read_csv ("simdat.csv", sep= ",")

sns.barplot(x = "group", y = "score", data = df, capsize= .1)




**Figure:**
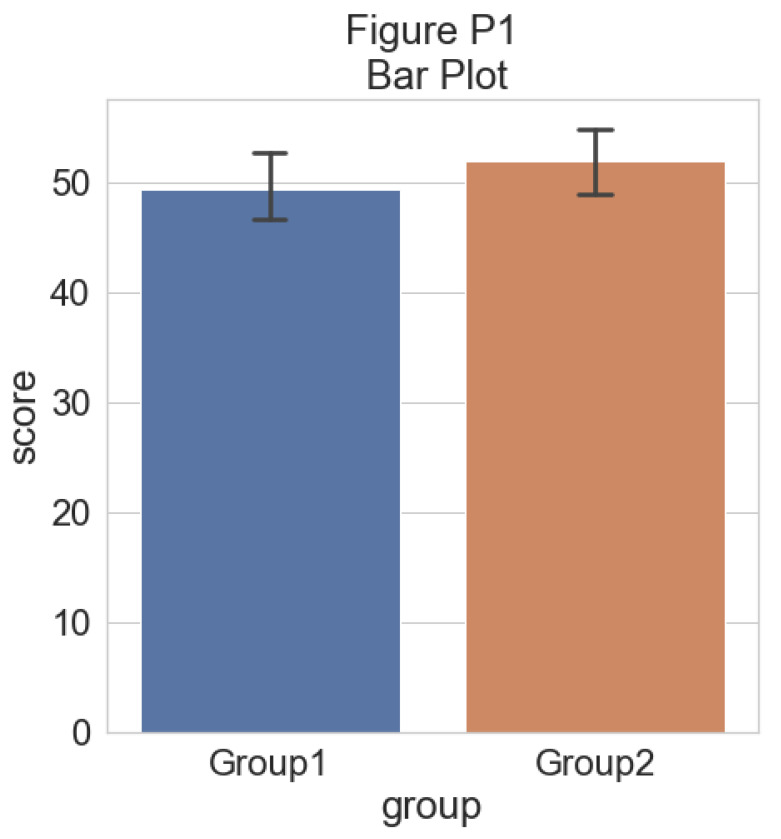


This plot can give the reader a first idea of the dataset: which group has a larger mean value, and whether this difference is likely to be significant or not. Only the mean of each group score and the standard deviation is visualized in this plot.

To have an idea of the distribution of our dataset we can plot a “cloud”, a smoothed version of the histogram:


# plotting the clouds
f, ax = plt.subplots(figsize=(7, 5))
dy="group"; dx="score"; ort="h"; pal = sns.color_palette(n_colors=1)

ax=pt.half_violinplot( x = dx, y = dy, data = df, palette = pal,
      bw = .2, cut = 0.,scale = "area", width = .6, inner = None,
      orient = ort)




**Figure:**
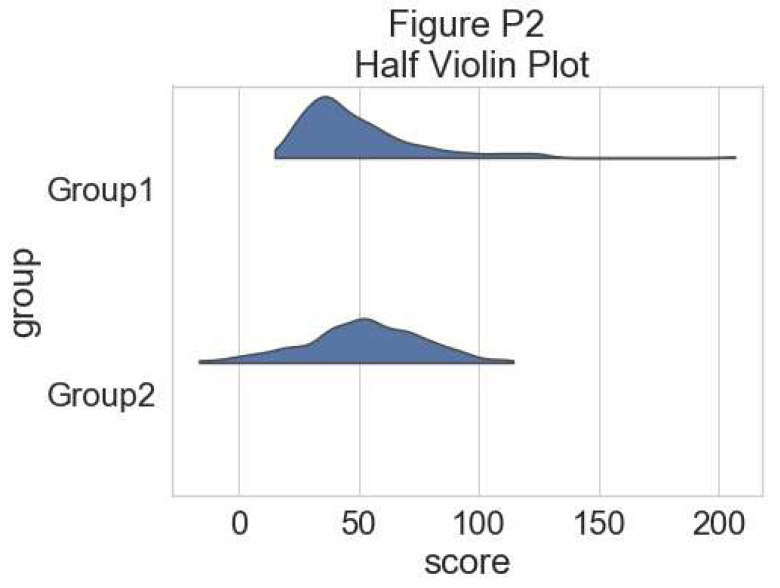


To have a more precise idea of the distribution and illustrate potential outliers or other patterns within the data, we now add the “rain”, a simple monodimensional representation of the data points:


# adding the rain
f, ax = plt.subplots(figsize=(7, 5))
ax=pt.half_violinplot( x = dx, y = dy, data = df, palette = pal,
       bw = .2, cut = 0.,scale = "area", width = .6, inner = None,
      orient = ort)

ax=sns.stripplot( x = dx, y = dy, data = df, palette = pal, 
     edgecolor = "white",size = 3, jitter = 0, zorder = 0,
     orient = ort)



**Figure:**
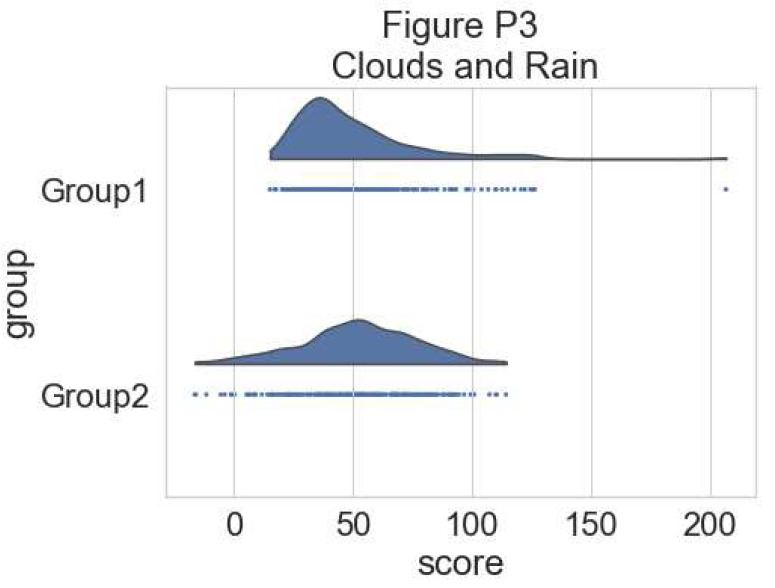



# adding jitter to the rain
f, ax = plt.subplots(figsize=(7, 5))
ax=pt.half_violinplot( x = dx, y = dy, data = df, palette = pal,
      bw = .2, cut = 0.,scale = "area", width = .6, inner = None, 
      orient = ort)

ax=sns.stripplot( x = dx, y = dy, data = df, palette = pal,
     edgecolor = "white",size = 3, jitter = 1, zorder = 0, 
     orient = ort)



**Figure:**
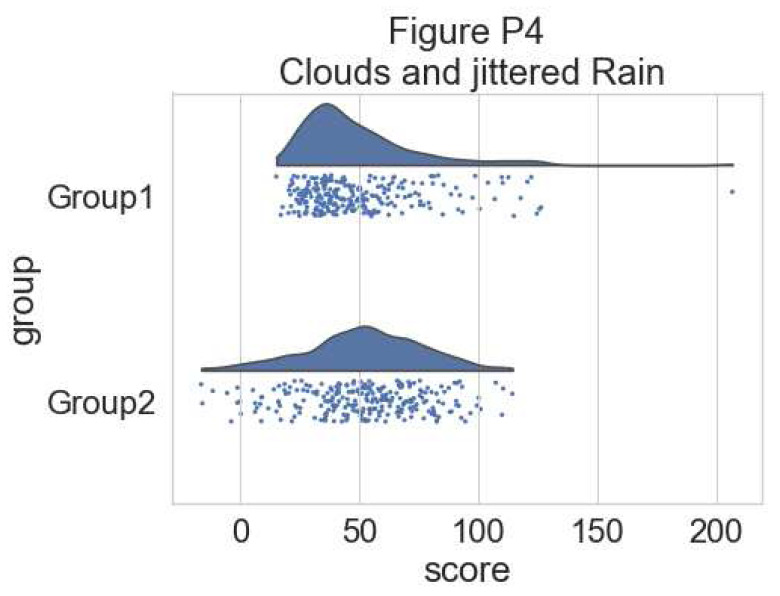


This gives a good idea of the distribution of the data points, but the median and the quartiles are not obvious, making it hard to determine statistical differences at a glance. Hence, we add an “empty” boxplot to show median, quartiles and outliers:


#adding the boxplot with quartiles
f, ax = plt.subplots(figsize=(7, 5))
ax=pt.half_violinplot( x = dx, y = dy, data = df, palette = pal,
       bw = .2, cut = 0.,scale = "area", width = .6, inner = None,
       orient = ort)

ax=sns.stripplot( x = dx, y = dy, data = df, palette = pal, 
       edgecolor = "white", size = 3, jitter = 1, zorder = 0, 
       orient = ort)

ax=sns.boxplot( x = dx, y = dy, data = df, color = "black", 
       width = .15, zorder = 10, showcaps = True,
       boxprops = {'facecolor':'none', "zorder":10}, showfliers=True,
       whiskerprops = {'linewidth':2, "zorder":10},
       saturation = 1, orient = ort)


**Figure:**
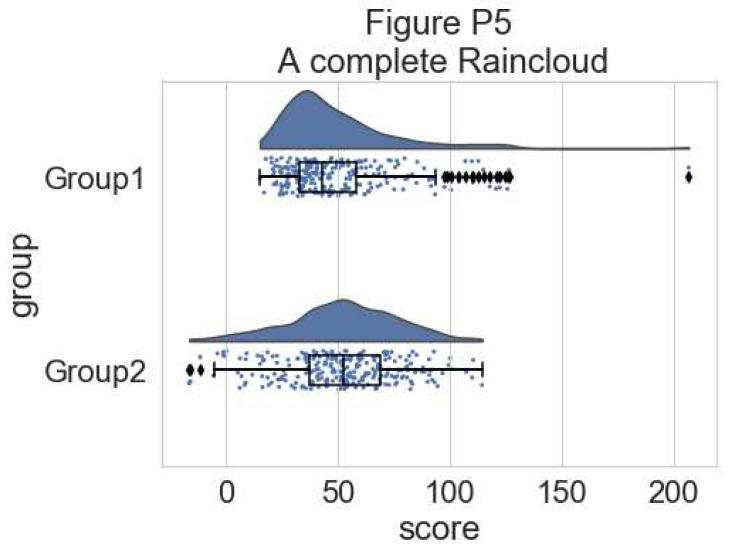


Now we can set a color palette to characterize the two groups:


#adding color
pal = "Set2"
f, ax = plt.subplots(figsize=(7, 5))
ax=pt.half_violinplot( x = dx, y = dy, data = df, palette = pal,
     bw = .2, cut = 0.,scale = "area", width = .6, 
     inner = None, orient = ort)

ax=sns.stripplot( x = dx, y = dy, data = df, palette = pal,
      edgecolor = "white",size = 3, jitter = 1, zorder = 0,
      orient = ort)

ax=sns.boxplot( x = dx, y = dy, data = df, color = "black",
      width = .15, zorder = 10, showcaps = True,
      boxprops = {'facecolor':'none', "zorder":10}, showfliers=True,
      whiskerprops = {'linewidth':2, "zorder":10}, 
      saturation = 1, orient = ort)



**Figure:**
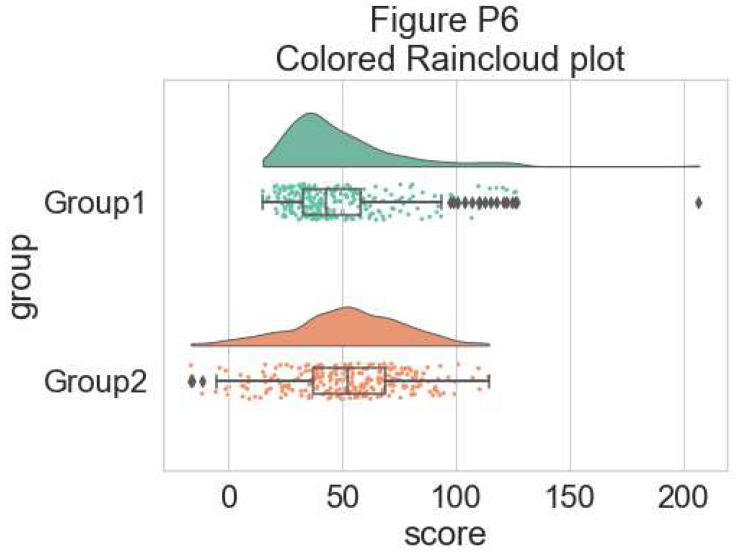


This plot is now both informative and aesthetically pleasing but written in far too many lines of code. We can use the function pt.Raincloud to add some automation:


#same thing with a single command: now x **must** be the categorical value
dx = "group"; dy = "score"; ort = "h"; pal = "Set2"; sigma = .2
f, ax = plt.subplots(figsize=(7, 5))

ax=pt.RainCloud(x = dx, y = dy, data = df, palette = pal,
      bw = sigma,width_viol = .6, ax = ax, orient = ort)



**Figure:**
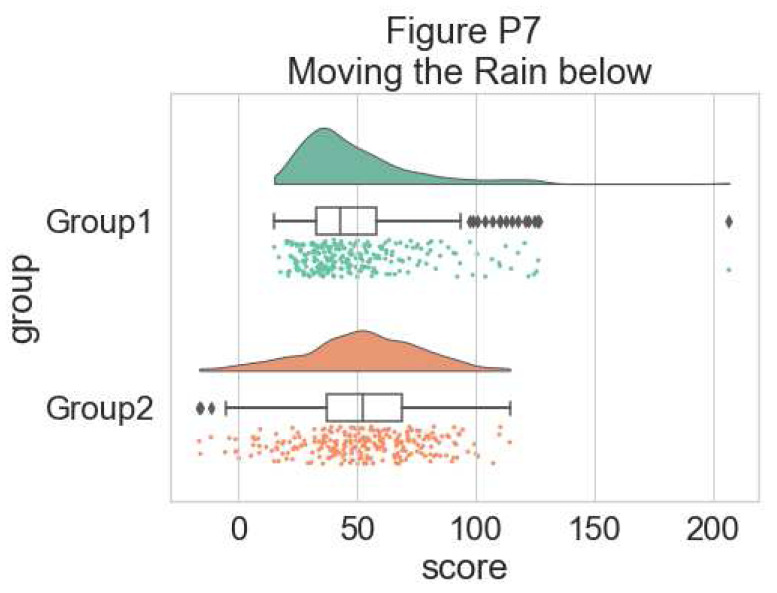


The ‘move’ parameter can be used to shift the rain below the boxplot, giving better visibility of the raw data in some instances:


#moving the rain below the boxplot
dx = "group"; dy = "score"; ort = "h"; pal = "Set2"; sigma = .2
f, ax = plt.subplots(figsize=(7, 5))

=pt.RainCloud(x = dx, y = dy, data = df, palette = pal, 
     bw = sigma, width_viol = .6, figsize = (7,5), 
     orient = ort, move = .2)


**Figure:**
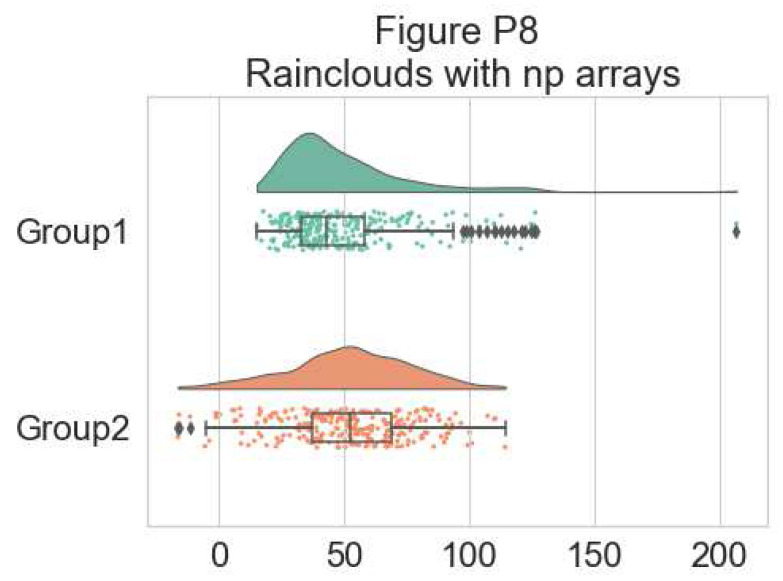


Further, the raincloud function works equally well with a list or numpy.array, if you prefer to use those instead of a dataframe input:


# Usage with a list/np.array input
dx = list(df["group"]); dy = list(df["score"])
f, ax = plt.subplots(figsize=(7, 5))

=pt.RainCloud(x = dx, y = dy, palette = pal, bw = sigma, 
      width_viol = .6, ax = ax, orient = ort)



**Figure:**
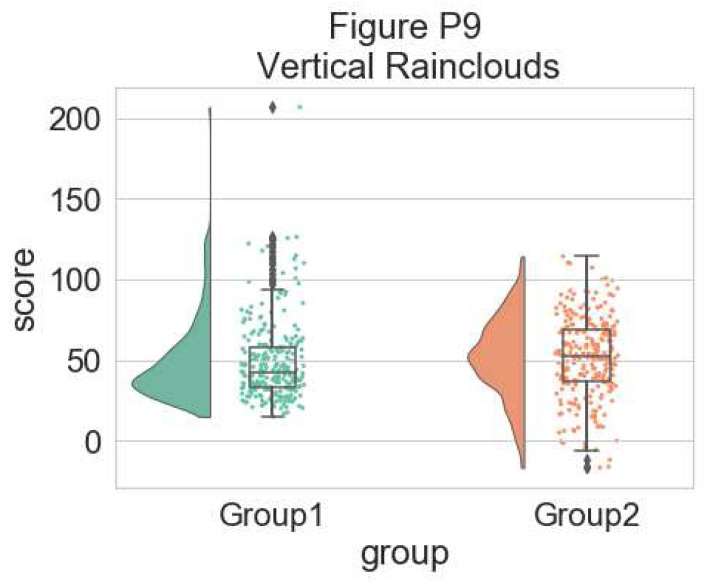


For some data, you may want to flip the orientation of the raincloud to a ‘petit prince’ plot. You can do this with the ‘orient’ flag in the pt.RainCloud Function:


# Changing orientation
dx="group"; dy="score"; ort="v"; pal = "Set2"; sigma = .2

f, ax = plt.subplots(figsize=(7, 5))
pt.RainCloud(x = dx, y = dy, data = df, palette = pal,
       bw = sigma,width_viol = .5, ax = ax, orient = ort)



**Figure:**
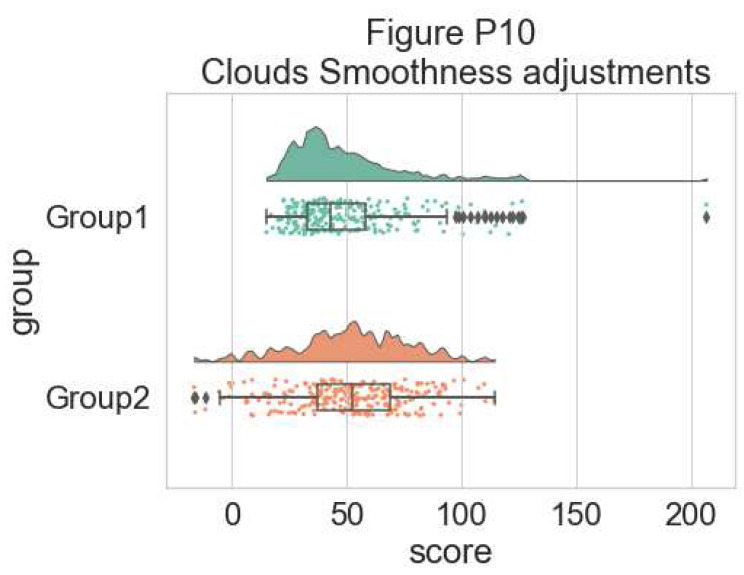


You can also change the smoothing kernel used to generate the probability distribution function of the data. To do this, you adjust the sigma parameter:


#changing cloud smoothness
dx="group"; dy="score"; ort="h"; pal = "Set2"; sigma = .05
f, ax = plt.subplots(figsize= (7, 5))

pt.RainCloud(x = dx, y = dy, data = df, palette = pal,
      bw = sigma,width_viol = .6, ax = ax, orient = ort)



**Figure:**
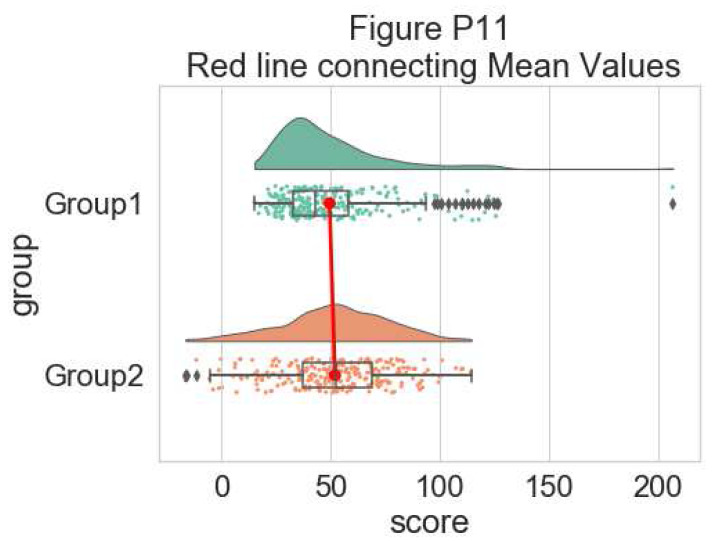


Finally, using the pointplot flag you can add a line connecting group mean values. This can be useful for more complex datasets, for example repeated measures or factorial data. Below we illustrate a few different approaches to plotting such data using rainclouds, by changing the hue, opacity, or dodge element of the individual plots:


#adding a red line connecting the groups' mean value (useful for longitudinal data)
dx="group"; dy="score"; ort="h"; pal = "Set2"; sigma = .2

f, ax = plt.subplots(figsize=(7, 5))
pt.RainCloud(x = dx, y = dy, data = df, palette = pal,
   bw = sigma, width_viol = .6, ax = ax, orient = 
    ort, pointplot = True)



**Figure:**
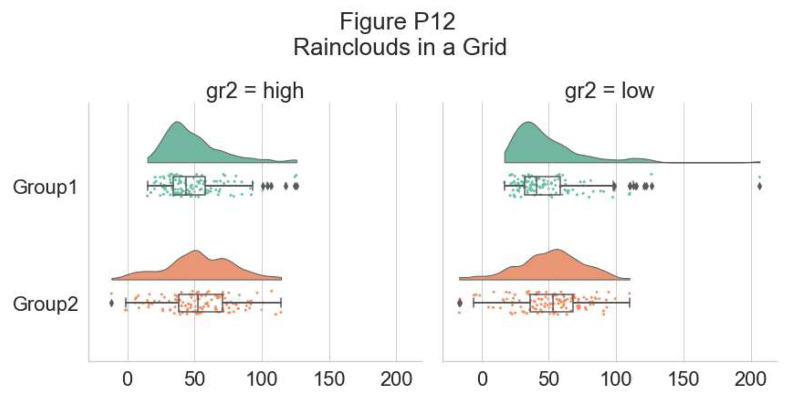


Another flexible option is to use Facet Grids to separate different groups or factor levels, illustrated below:


# Rainclouds with FacetGrid
g = sns.FacetGrid(df, col = "gr2", height = 6)
g = g.map_dataframe(pt.RainCloud, x = "group", y = "score",
       data = df, orient = "h")
g.fig.subplots_adjust(top = 0.75)


**Figure:**
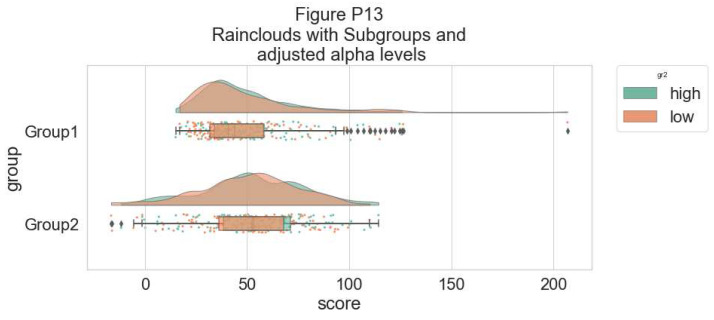


As an alternative, it is possible to use the hue input for plotting different sub-groups directly over one another, facilitating their comparison:


# Hue Input for Subgroups
dx="group"; dy="score"; dhue="gr2"; ort="h" pal="Set2"; sigma = .2

f, ax = plt.subplots(figsize=(12, 5))
pt.RainCloud(x = dx, y = dy, hue = dhue, data = df,
      palette = pal, bw = sigma,width_viol = .7, ax = ax,
      orient = ort)


**Figure:**
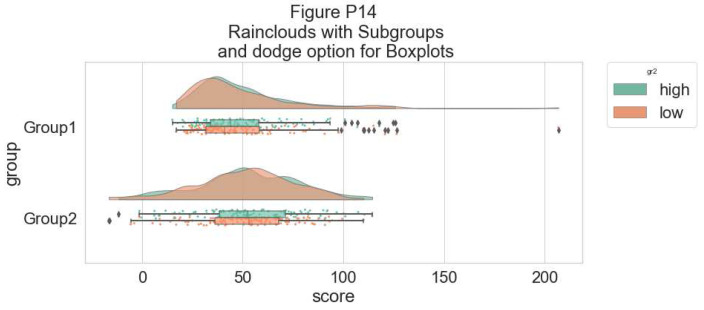


To improve the readability of this plot, we adjust the alpha-level using the associated flag (0–1 alpha intensity):


# Setting alpha level
f, ax = plt.subplots(figsize=(12, 5))
pt.RainCloud(x = dx, y = dy, hue = dhue, data = df,
      palette = pal, bw = sigma, width_viol = .7, ax = ax, 
      
      orient = ort , alpha = .65)


**Figure:**
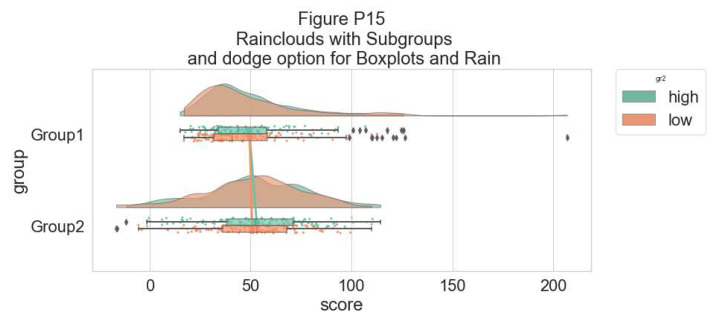


Rather than letting the two boxplots obscure one another, we can set the dodge flag to true, adding interpretability:


#The Dodge Flag
f, ax = plt.subplots(figsize=(12, 5))
pt.RainCloud(x = dx, y = dy, hue = dhue, data = df,
      palette = pal, bw = sigma,width_viol = .7, ax = ax,
      
      orient = ort , alpha = .65, dodge = True)


**Figure:**
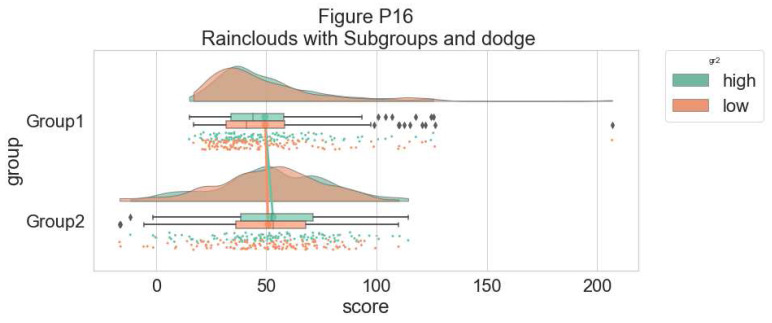


Finally, we may want to add a traditional line-plot to our graph to aid in the detection of factorial main effects and interactions. As an example, we’ve plotted the mean within each boxplot:


#same, with dodging and line
f, ax = plt.subplots(figsize=(12, 5))

pt.RainCloud(x = dx, y = dy, hue = dhue, data = df,
     palette = pal, bw = sigma, width_viol = .7, ax = ax,
      orient = ort , alpha = .65, dodge = True, pointplot = True)


**Figure:**
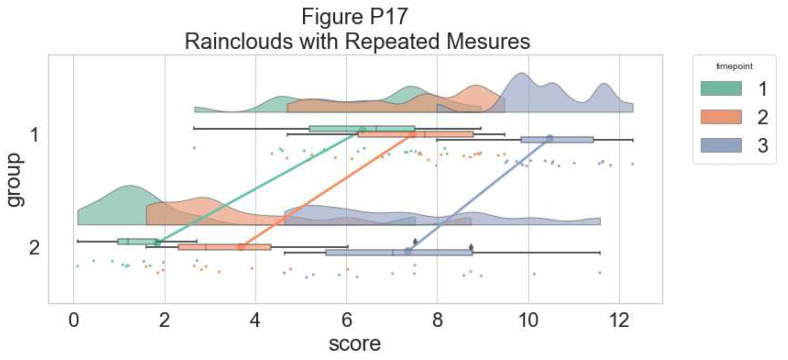


Here is the same plot, but now with the individual observations moved below the boxplots again using the ‘move’ parameter:


#moving the rain under the boxplot 

f, ax = plt.subplots(figsize=(12, 5))
pt.RainCloud(x = dx, y = dy, hue = dhue, data = df, 
     palette = pal, bw = sigma, width_viol = .7, ax = ax,
     
      orient = ort , alpha = .65, dodge = True, pointplot = True,
     move = .2)


**Figure:**
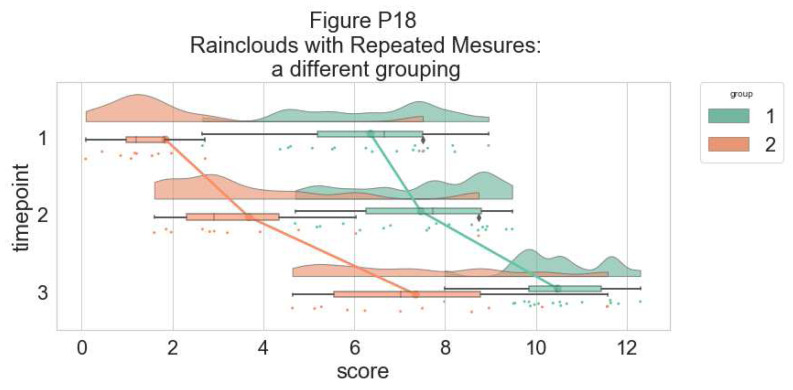


As our last example, we’ll consider a complex repeated measures design with two groups and three timepoints. The goal is to illustrate our complex interactions and main-effects, while preserving the transparent nature of the raincloud plot:


# Load in the repeated data
df_rep = pd.read_csv ("repeated_measures_data.csv", sep= ",",
        header = None)
df_rep.columns = ["score",  "timepoint", "group"]

# Plot the repeated measures data
dx = "group"; dy="score"; dhue="timepoint"
ort="h"; pal="Set2"; sigma = .2
f, ax = plt.subplots(figsize=(12, 5))

pt.RainCloud(x = dx, y = dy, hue = dhue, data = df_rep,
     palette = pal, bw = sigma, width_viol = .7, ax = ax, 
      orient = ort , alpha = .65, dodge = True, pointplot = True, 
     move = .2)


**Figure:**
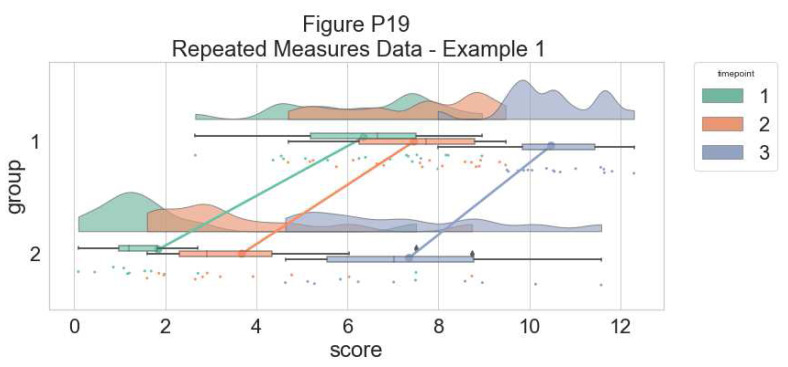


The function is flexible enough that you can flip the ordering of the factors around simply by changing which variable informs the hue parameter:


# Now with the group as hue
dx = "timepoint"; dy = "score"; dhue = "group"
f, ax = plt.subplots(figsize=(12, 5))

pt.RainCloud(x = dx, y = dy, hue = dhue, data = df_rep, 
      palette = pal, bw = sigma, width_viol = .7, ax = ax,
      
      orient = ort, alpha = .65, dodge = True, pointplot = True,
      move = .2)


**Figure:**
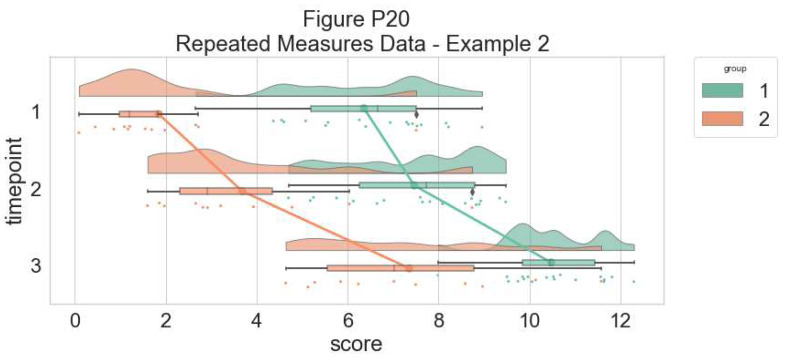


That’s it! Hopefully this tutorial has given you an idea of some of the different ways you can produce raincloud plots in Python. Next, we’ll describe how to produce these plots in Matlab.

### How to Make it Rain in Matlab

Matlab (Mathworks Inc.) is a proprietary mathematical programming language used widely in engineering, the physical sciences, and neuroscience. The code for this tutorial can be found at:

https://github.com/RainCloudPlots/RainCloudPlots/tree/master/tutorial_matlab

Here you can also find functions to create raincloud-plots ( raincloud_plot.m and rm_raincloud.m), as well as a “live notebook” ( raincloud_plots_tutorial.mlx) which walks the user through the customization of various raincloud plots.

First, we’ll set up our path and use the colorbrewer function to define some nice colour palettes:


% set up a dynamic path
% script must be run from parent directory containing all three tutorial
% directories (i.e., the one 'above' the directory 'tutorial_matlab')

pardir = pwd;
figdir = fullfile(pardir, 'figs', 'tutorial_matlab');
if ~exist('figdir', 'dir')
     mkdir(figdir);
end

% make sure functions to generate plots are on the path
codedir = fullfile(pardir, 'tutorial_matlab');
addpath(codedir);

try
     % get nice colours from colorbrewer
     % (https://uk.mathworks.com/matlabcentral/fileexchange/34087-cbrewer---colorbrewer-schemes-for-matlab)
     [cb] = cbrewer('qual', 'Set3', 12, 'pchip');
catch
     % if you don't have colorbrewer, accept these far more boring colours
     cb = [0.5 0.8 0.9; 1 1 0.7; 0.7 0.8 0.9; 0.8 0.5 0.4; 0.5 0.7 0.8; 1 0.8 0.5; 0.7 1 0.4; 1 0.7 1; 0.6 0.6 0.6; 0.7 0.5 0.7; 0.8 0.9 0.8; 1 1 0.4];
end

cl(1, :) = cb(4, :);
cl(2, :) = cb(1, :);

fig_position = [200 200 600 400]; % coordinates for figures



Now we’ll generate some datapoints with similar means and standard deviations; the first is drawn from a random normal distribution and the second from a random exponential distribution. We’ll plot these same data repeatedly in different ways further down:


n = 250;

% set a random number generator seed for reproducible results
rng(123)

d{1} = [exprnd(5, 1, n) + 15]';
d{2} = [(randn(1, n) *5) + 20]';

means = cellfun(@mean, d);
variances = cellfun(@std, d);



Let’s create a quick bar graph of these data. This is the kind of standard visualization you see in many papers, depicting the mean of the data plus standard deviation:


f1 = figure('Position',fig_position); hold on;
h = bar(means, 'FaceColor', 'flat', 'LineWidth',.9);

h(1).CData(1, :) = cl(1, :);
h(1).CData(2, :) = cl(2, :);

e = errorbar(1:2, means, variances, '.k', 'LineWidth',.9);
set(gca, 'XTick', 1:2)
title('Bar Plot');

% save
print(f1, fullfile(figdir, '1bar.png'), '-dpng');



**Figure:**
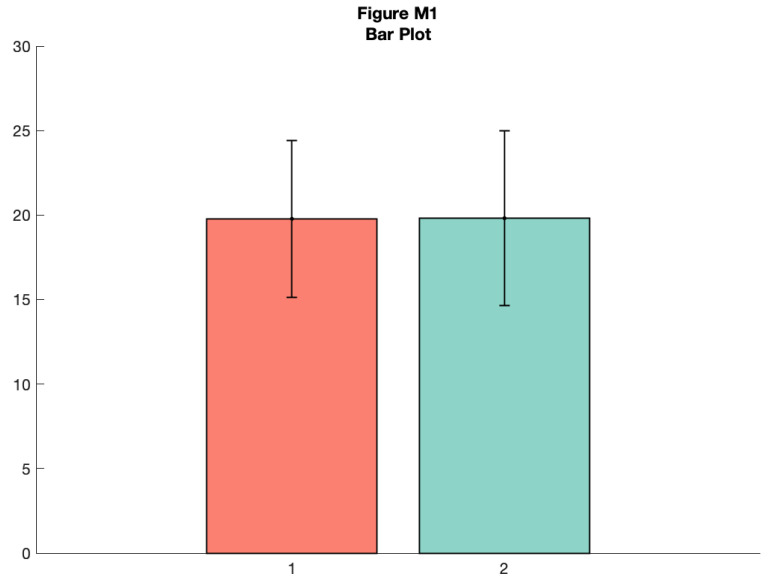


As you can see, this tells you something about the data, but a lot of really useful and important information is hidden such as the ‘shape’ or distribution of the data and the raw observations themselves. A histogram nicely shows some of what we’re missing:


f2 = figure('Position', fig_position);
subplot(1, 2, 1)
[n1, x1] = hist(d{1}, 30);
bar(x1, n1, 'FaceColor', cl(1,:), 'EdgeColor', 'k');
title('Histogram') 
subplot(1, 2, 2)
[n2, x2] = hist(d{2}, 30);
bar(x2, n2, 'FaceColor', cl(2,:), 'EdgeColor', 'none');

% save
print(f2, fullfile(figdir, '2hist.png'), '-dpng');



**Figure:**
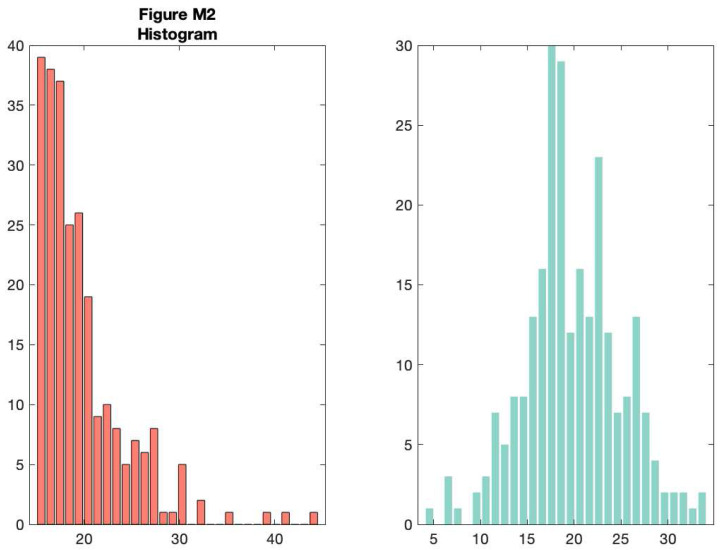


However, now we’ve lost the summary data. The raincloud plot tries to bring these elements together in one intuitive plot. You can use the ‘raincloud_plot.m’ function accompanying this tutorial to produce these plots in Matlab:


f3 = figure('Position', fig_position);
subplot(2, 1, 1)
h1 = raincloud_plot('d{1}, 'box_on', 1);
title('Raincloud Plot: Group 1')
set(gca,'XLim', [0 40]);
box off
subplot(2, 1, 2)
h2 = raincloud_plot(d{2}, 'box_on', 1);
title('Raincloud Plot: Group 2');
set(gca,'XLim', [0 40]);
box off



% save
print(f3, fullfile(figdir, '3Rain1.png'), '-dpng');




**Figure:**
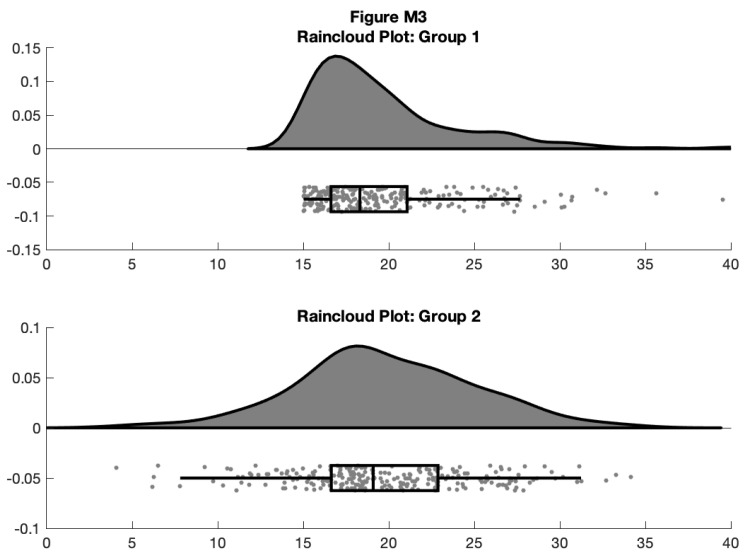


This gives us the distribution (probability density plot), summary data (box plot), and raw observations all in one place. Now we’ll walk you through some of the options of the function, which you can use to change various aesthetic properties of the plot. The function only requires a vector of the data you want to plot as the input. Additionally, there are a variety of optional flags you can call to turn the boxplots on and off, to alter ('dodge') the position of the boxes and dots, and to change various aesthetics such as linewidth, colors, and so on. For example, by setting a few different flags we can create more colorful plots:


f4 = figure('Position', fig_position);
subplot(2, 1, 1)
h1 = raincloud_plot(d{1}, 'box_on', 1);
title('Raincloud Plot: Default Plot')
set(gca,'XLim', [0 40]);
box off
subplot(2, 1, 2)
h2 = raincloud_plot(d{1}, 'box_on', 1, 'box_dodge', 1, 'box_dodge_amount',...
0, 'dot_dodge_amount', .3, 'color', cb(1,:), 'cloud_edge_col', cb(1,:));
title('Raincloud Plot: Some Aesthetic Options');
set(gca,'XLim', [0 40]);
box off

% save
print(f4, fullfile(figdir, '4Rain2.png'), '-dpng');




**Figure:**
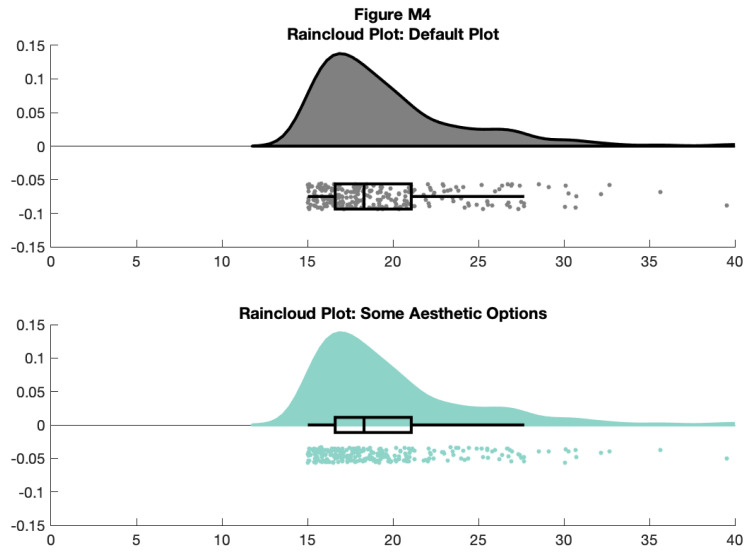


The function returns a cell array for various figure parts, so you can also call the base function and then change things with normal ‘set’ commands, like so:


f5 = figure('Position', fig_position);
subplot(2, 1, 1)
h1 = raincloud_plot(d{1}, 'box_on', 1);
title('Raincloud Plot: Default Plot')
set(gca,'XLim', [0 40]);
box off
subplot(2, 1, 2)
h2 =  raincloud_plot(d{1}, 'box_on', 1);
title('Raincloud Plot: Some Aesthetic Options');
set(h2{1},'FaceColor', cb(1, :)) % handles 1-6 are the cloud area,
scatterpoints, and boxplot elements respectively
set(h2{2}, 'MarkerEdgeColor', 'red') % 
set(gca,'XLim', [0 40]);
box off

% save
print(f5, fullfile(figdir, '5Rain3.png'), '-dpng');


**Figure:**
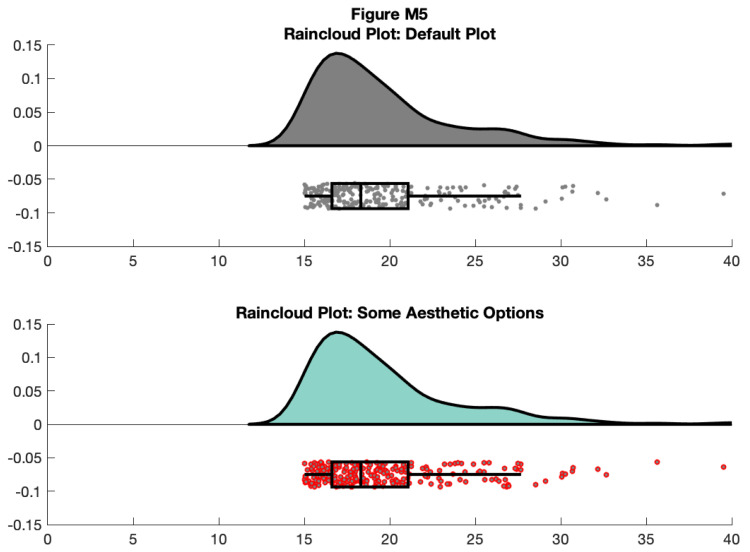


You can also control the smoothness of the probability density function by calling the ‘bandwidth’ parameter. Additionally, if you have Cyril Pernet’s robust statistics toolbox on your path, you can call the ‘rash’ function for an alternative kernel density function:


f6 = figure('Position', fig_position);
subplot(2, 1, 1)
h1 = raincloud_plot(d{1}, 'box_on', 1, 'color', cb(1,:), 'bandwidth', .2,
'density_type', 'ks');
title('Raincloud Plot: Reduced Smoothing, Kernel Density')
set(gca,'XLim', [0 40]);
box off
subplot(2,1,2)
h2 = raincloud_plot(d{1}, 'box_on', 1, 'color', cb(2,:), 'bandwidth', 1,
'density_type', 'rash');
title('Raincloud Plot: Rash Density Estimate')
set(gca,'XLim', [0 40]);
box off

% save
print(f6, fullfile(figdir, '6Rain4.png'), '-dpng');


**Figure:**
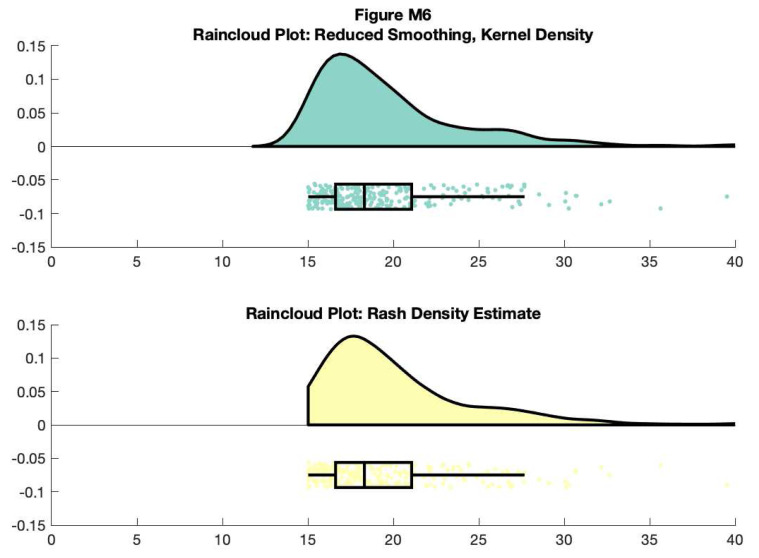


Here, we’ll use the dot and box dodge options to create an overlapping set of raincloud plots, useful for group comparison. The function can be called repeatedly (e.g., from within a loop) - each iteration will overlay the previous. Note that here we’re using the ‘alpha’ parameter to make the plot area see-through:


% example 1
f7 = figure('Position', fig_position);
subplot(1, 2 ,1)
h1 = raincloud_plot(d{1}, 'box_on', 1, 'color', cb(1,:), 'alpha', 0.5,...
    'box_dodge', 1, 'box_dodge_amount', .15, 'dot_dodge_amount', .15,...
    'box_col_match', 0);
h2 = raincloud_plot(d{2}, 'box_on', 1, 'color', cb(4,:), 'alpha', 0.5,...
    'box_dodge', 1, 'box_dodge_amount', .35, 'dot_dodge_amount', .35, 'box_col_match', 0);
legend([h1{1} h2{1}], {'Group 1', 'Group 2'})
title('A) Dodge Options Example 1')
set(gca,'XLim', [0 40], 'YLim', [-.075 .15]);
box off

% example 2
subplot(1, 2, 2)
h1 = raincloud_plot(d{1}, 'box_on', 1, 'color', cb(1,:), 'alpha', 0.5,...
    'box_dodge', 1, 'box_dodge_amount', .15, 'dot_dodge_amount', .35,...
    'box_col_match', 1);
h2 = raincloud_plot(d{2}, 'box_on', 1, 'color', cb(4,:), 'alpha', 0.5,...
    'box_dodge', 1, 'box_dodge_amount', .55, 'dot_dodge_amount', .75,...
    'box_col_match', 1);
legend([h1{1} h2{1}], {'Group 1', 'Group 2'})
title('B) Dodge Options Example 2')
set(gca,'XLim', [0 40]);
box off

% save
print(f7, fullfile(figdir, '7Rain5.png'), '-dpng');



**Figure:**
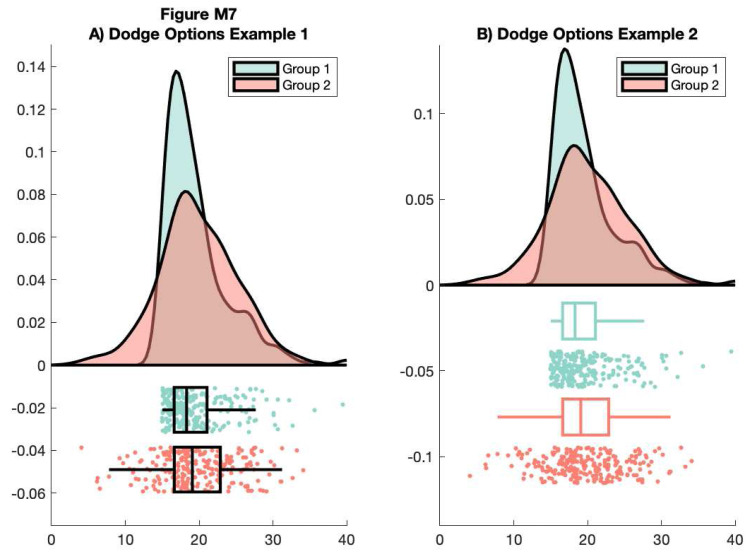


You can control the jitter and position of the ‘raindrops’ in the Y-plane by calling the figure handles:


f8 = figure('Position', fig_position);
subplot(2, 1, 1)
h1 = raincloud_plot(d{1}, 'color', cb(5,:));
set(gca,'XLim',[0 40]);
h1{2}.YData = repmat(-0.1, n, 1);

subplot(2, 1, 2)
h2 = raincloud_plot(d{2}, 'color', cb(7,:));
set(gca,'XLim',[0 40]);
h2{2}.YData = repmat(-0.05,n,1); 

% save
print(f8, fullfile(figdir, '8Rain6.png'), '-dpng');


**Figure:**
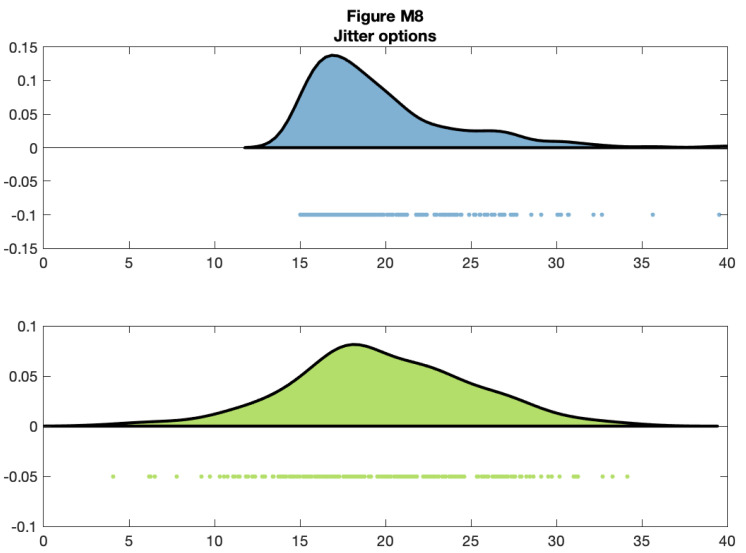


For the final examples, we'll consider a more complex factorial situation where we have multiple groups and observations. To illustrate this, we'll use a more complex implementation of rainclouds encoded in the 'rm_raincloud.m' function.


% grab 'repeated_measures_data.csv';
D = dlmread(fullfile(codedir, 'repeated_measures_data.csv'));

% read into cell array of the appropriate dimensions
for i = 1:3
    for j = 1:2
        data{i, j} = D(D(:, 2) == i & D(:, 3) ==j);
    end
end

% make figure
f9  = figure('Position', fig_position);
h   = rm_raincloud(data, cl);
set(gca, 'YLim', [-0.3 1.6]);
title('repeated measures raincloud plot');

% save
print(f9, fullfile(figdir, '9RmRain1.png'), '-dpng');


**Figure:**
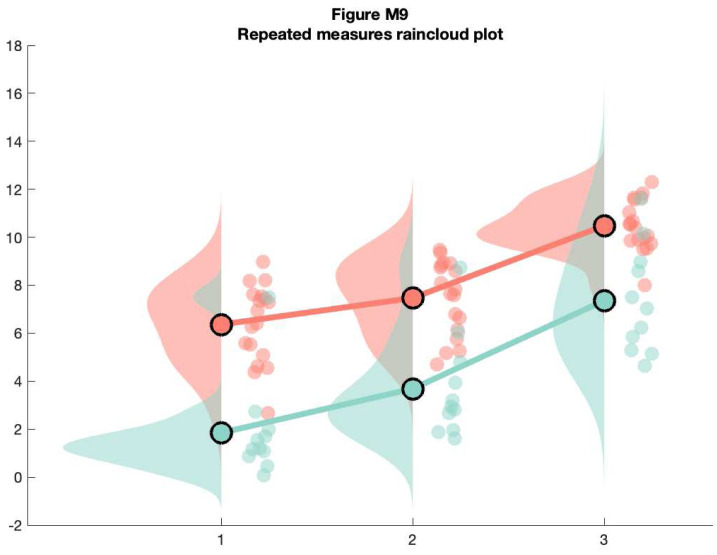


 As above, 'rm_raincloud.m' returns a cell-array of handles to the various figure parts. We can add aesthetic options by calling these handles.


% make figure
f10 = figure('Position', fig_position);
h   = rm_raincloud(data, cl);
set(gca, 'YLim', [-0.3 1.6]);
title('repeated measures raincloud plot - some aesthetic options')

% define new colour
new_cl = [0.2 0.2 0.2];

% change one subset to new colour and alter dot size
h.p{2, 2}.FaceColor         = new_cl;
h.s{2, 2}.MarkerFaceColor   = new_cl;
h.m(2, 2).MarkerEdgeColor   = 'none';
h.m(2, 2).MarkerFaceColor   = new_cl;
h.s{2, 2}.SizeData          = 300;

% save
print(f10, fullfile(figdir, '10RmRain2`.png'), '-dpng');


**Figure:**
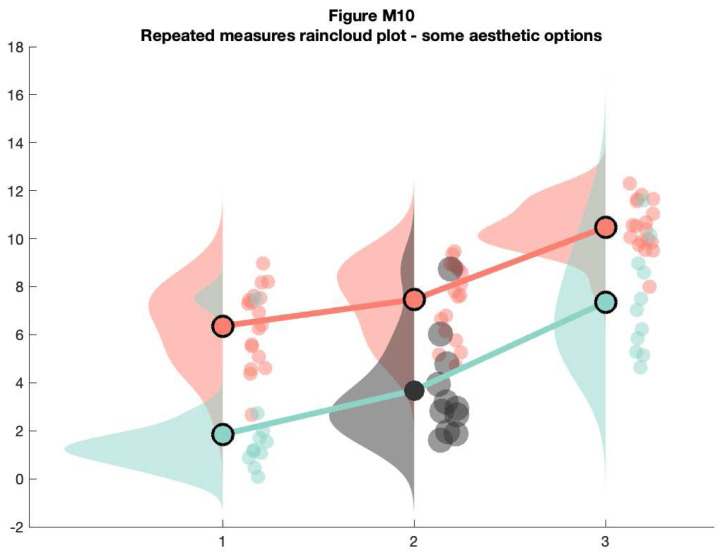


That’s it! Now you should be ready to customize your Raincloud plots for a variety of different purposes. This concludes our cross-platform tutorial!

## Discussion

We hope that our tutorials demonstrate the flexibility of raincloud plots for visualizing data. Raincloud plots build on a rich tradition of data graphics, enabling the user to visualize key parameters for statistical inference in a transparent an aesthetically appealing fashion. In this sense, Rainclouds are part of a wider family of plotting tools such as beeswarms ( Eklund, 2016), strip plots ( Tukey, 1970), and estimation plots ( Ho *et al*., 2018).

Indeed, our goal is not to argue for the superiority or novelty of raincloud plots over these and other complementary methods. Our focus is on providing a robust cross-platform tool for creating transparent plots. In general, the modularity of the raincloud plot is a strength, and we encourage the user to think carefully about the choice of individual elements (clouds, rain, & confidence intervals) depending on the particularities of their data.

It is worth mentioning that here we envision these three aspects of the raincloud plots as sub-serving particular statistical goals. In our examples, the probability distributions depicted by the split-half violin plot (‘clouds’) illustrate the sample variance. As such they are excellent tools for assessing how data are distributed and checking assumptions (i.e., violations of normality). Considering this, we caution against the use of clouds in this form for statistical inference at a glance, which is better served by comparing some parameter estimates in relation to their uncertainty. Users who wish to use probability distributions for inference should instead consider a more suitable approach such as estimation plots, or by plotting a smoothed histogram of bootstrapped parameter estimates, or simply by plotting rainclouds with boxplots and/or confidence intervals, as we have done in our tutorial examples. The code provided with this tutorial makes it easy to implement whatever histogram function best suits the needs of the user, simply by substituting the PDF estimation function.

Additionally, at first glance it may seem redundant to plot both raw datapoints (‘rain’) and data distributions (‘clouds’). However, we put forth that plotting both offers several advantages. First, plotting raw datapoints can enable the automated (i.e., machine-readable) recovery of data from plots even when the data underlying the plot has been lost. Second, plotting raw data can facilitate the identification of unexpected patterns within the data, such as ordinality or outliers, which may not be readily apparent from a probability distribution or box-plot alone. As such we recommend the combination of raw data plots and smoothed distributions (however estimated) wherever possible.

In the spirit of open science and supporting each other in improving our data visualisations, we invite readers to contribute their own variations and extensions directly to our GitHub repository ( https://github.com/RainCloudPlots/RainCloudPlots). Directions on how to contribute can be found in our contributing guidelines. We are particularly indebted to the Binder team ( Jupyter *et al*., 2018), part of Project Jupyter ( http://jupyter.org), whose tool allows all users to explore the R and Python examples interactively from the browser.

### Preprints, Pull Requests and the value of community science

This manuscript was originally published as a preprint on the Peerj platform ( https://doi.org/10.7287/peerj.preprints.27137v1). The eight months since have illustrated the remarkable potential of new publishing infrastructure and landscape make the process of publishing scientific content faster, better and more collaboratively. We here outline just a few of the positives from doing so, and hope this may serve to encourage others. Firstly, posting the manuscript as preprint has vastly widened the reach. To date (March 2019) our preprint was viewed 9803 times, with 6309 downloads. However, views and downloads alone don’t necessarily entail engagement. Since publication the preprint alone has already been cited 18 times. Moreover, in depth engagement has gone well beyond mere citations. Several individuals have created their own useful tutorials, summarizing our paper and asking useful questions, posted constructive criticism, discussed raincloud plots as part of various plotting alternatives, created a shiny app, wrote an accessible tutorial using native R datasets, a new package, creating various animated interactive visualisations (github here), used to illustrate the Binder format and used in more informal blogposts on e.g. superforecasting. Our codebase itself received feedback through various avenues including formal pull requests on github, comments on the preprint, twitter replies and email. In this new version of our paper we have tried our best to integrate all these suggestions and comments, which without fail have improved the usability of our code.

Social media, specifically twitter, provided the central hub where all these benefits coalesced. The paper has been tweeted at least 750 times, with an estimated reach of up to 1,500,000 total followers, and as such is the principal driver for the engagement our preprint has received. This engagement has yielded invaluable feedback, comments, and suggestions, and were even lucky enough to track down the first instance of an early precursor of the raincloud plot ( Ellison, 1993). Moreover, the paper itself was inspired by a twitter discussion, and brings together co-authors who have never met in person. Together, these interactions illustrate the fundamentally two-way street of new publishing models, which facilitate access without paywalls and allow for near instantaneous improvements to ongoing work.

## Conclusion

The future of data science lies in reproducible, robust methods that communicate our results to as wide of an audience as possible. We hope that raincloud plots will help you to better understand and communicate your own data-analysis. In the present paper, we’ve outlined some of the strengths of these plots compared to traditional methods such as bar or violin-plots. Using the attached code and tutorials, this paper opens up the raincloud plot to a wide variety of scientists in a multitude of disciplines.

## Software availability

Code available from: https://github.com/RainCloudPlots/RainCloudPlots

Archived code as at time of publication: http://doi.org/10.5281/zenodo.1402959 ( Allen *et al*., 2018).

License: MIT

## Footnotes

^1^ This raises the question of why such uninformative plots became widespread in the first place. Speculatively, they may simply have been easier to produce before the advent of personal computers and associated statistical software, when plots were typically hand-drawn. Manual plotting of this type was time consuming and error-prone; simply plotting all raw data points would have considerably increased workload and the full-scale plotting of probability distributions may have been beyond the grasp of many researchers.

^2^ Indeed, try it yourself at http://guessthecorrelation.com/

^3^ See http://www.fharrell.com/post/interactive-graphics-less/ for an interactive demonstration of how raincloud-like plots can aid minimal yet powerful inference.
