# Supplementary material for: Raincloud plots: a multi-platform tool for robust data visualization
Source: Wellcome Open Res. 2021 Jan 21;4:63. Originally published 2019 Apr 1. [Version 2] doi: 10.12688/wellcomeopenres.15191.2 (PMC6480976; doi:10.12688/wellcomeopenres.15191.2)
Supplement: Peer review history

# Reviewer report: Reviewer response for version 1

**DOI:** 10.21956/wellcomeopenres.16574.r35184

**Published:** 2019-04-17

**Authors:** Elena Allen

**Affiliations:** 1 Rodin Scientific, LLC, Albuquerque, MN, USA

The authors present a software tool to facilitate the creation of more informative data visualizations. Specifically, the "raincloud" plot offers accessibility to distributional shape, statistical summaries, and individual data points. What more could you want? While the ideas underlying the raincloud aren't novel (and the authors do address violins, boxes, beans, rugs, strips, swarms, and other predecessors), the paper provides an excellent tutorial for the uninitiated who may be unfamiliar with better approaches to data visualization. It's one thing to point out the shortcomings of standard plots and decry, "we need to do better!"; it's quite another to provide a tool that gets the job done in a few lines of code. I am particularly impressed with the thoroughness of the code examples and the commitment of the authors to make their tools open and available to the largest possible audience. Moreover, the inclusion of R, Python, and Matlab will make almost everyone happy. Folks can get started with their favorite flavor right away. Well done.  

 I have only relatively minor comments and suggestions for revision.

 1. In the Discussion, I'd like the authors to more fully address the downsides of a raincloud plot (every plot has them). Yes, you are adding information, but at what cost? Some things that come to mind: space and ink. 

 Space is always at a premium in publications and presentations of dense/high-dimensional datasets. When is the inclusion of a cloud, rain and summary stats justified? How might this depend on the user's **goals **in creating the visualization, rather than just the "particularities of their data". Also, does the separation between groups necessitated by the layout of the raincloud hamper a human's ability to make comparisons? For two-group comparisons, I've always found the "asymmetric violin" (e.g., Fig. 4 of Kampstra's Beanplot paper ^1^) to be unmatched in it's ability to encourage comparison. How does a raincloud compare? 

 Likewise, it takes a good amount of ink to create clouds; if you've already displayed all the individual data points and an indication of centrality or statistical uncertainty, does the cloud give you anything new? In some cases it would seem to violate Tufte's guideline for data-ink ratio minimization. For me, the desired complexity of the visualization and utility of different aspects is often a function of dataset size. For example, in Figure R10 the clouds are very pretty but I already knew everything I needed to know from the relatively few data points and boxplots. In fact, one could argue that there are too few points to support such kernel density estimates. A cloud might by more appropriate in a mid-size dataset (e.g., Figure R9) where one has difficultly estimating distributional shape by eye (of course, a simple beeswarm visualization addresses this limitation in full). When the dataset gets very large the raindrops (as instantiated here) become useless. The authors do "encourage the user to think carefully about the choice of individual elements", but I'd like them to go further and identify cases where aspects of the raincloud plot might be more or less useful.

 2. A comment on footnote 1, regarding the widespread use of box plots: while I can fully agree that bar plots are over- and inappropriately used, the authors (or at least the founders of #barbarplots) seem to have forgotten that bar plots are an appropriate and intuitive visualization for counts, proportions, and frequencies, where there is no interesting distributional information to display and we really just care about how much or how often a thing happened. You could use a raincloud plot to display, e.g., the proportion of trials that were successful in a task, but this is just a distribution of 0's and 1's -- your cloud would be an odd one for sure, and the resulting visualization would be a non-intuitive representation of what you actually care about.

 3. A comment on the criticism of violin plots (p. 4) and associated footnote 4: Have the authors considered that if they centered their cloud PDFs, they would take up just as much ink as a violin?  I'm not sure "data-ink ratio" can be invoked here. Regarding "overly provocative" violin plots...I get that it's a funny comic and the authors are in on the joke. But this is a peer-reviewed tutorial article likely to be targeted at young, diverse scientists and researchers. The authors should hold themselves to a higher standard and perhaps reflect on the underlying and unintended misogyny that such a graphic perpetuates. Can violin plots look like vaginas? Sure. Can bar plots look like dicks? Yeah. Get over it and let's get back to work.

 4. A few nit-nats on the tutorial so that the visualizations follow best practices:

- In R11 and R12, avoid using a dotted or hashed line to connect repeated measures - it is very difficult to distinguish those dots from the actual data points in the raindrops.
- In example M6, yellow has virtually no contrast on white, so we really can't see the raindrops. A poor choice for color encoding.
- In the code for example M3, there is an extra apostrophe before d{1} in h1 = raincloud_plot('d{1}, 'box_on', 1);
- In all the Matlab examples please **label the axes**. Axes have been purposefully labeled as "Group" and "Score", or "Time" and "Group" in R but neglected in Matlab. As someone who spent a long time characterizing how frequently authors fail to label their variables ^2^, it makes me cringe to see the same mistake being made in a tutorial.
- In the code for M9, I would include comments to help readers understand how/why data is being re-packaged into a cell array. For example:

D = dlmread(fullfile(codedir, 'repeated_measures_data.csv'));

 % D is a structured as [value, time, group]

 % read into cell array of the appropriate dimensions

 nGroup = 2;

 nTime = 3;

 data = cell(nTime, nGroup);

 for ii = 1:nTime

     for jj = 1:nGroup

         data{ii, jj} = D(D(:, 2) == ii & D(:, 3) == jj);

     end

 end

Are the conclusions about the tool and its performance adequately supported by the findings presented in the article?

Yes

Is the rationale for developing the new software tool clearly explained?

Yes

Is the description of the software tool technically sound?

Yes

Are sufficient details of the code, methods and analysis (if applicable) provided to allow replication of the software development and its use by others?

Yes

Is sufficient information provided to allow interpretation of the expected output datasets and any results generated using the tool?

Yes

Reviewer Expertise:

Very experienced in data visualization with Matlab expertise.

I confirm that I have read this submission and believe that I have an appropriate level of expertise to confirm that it is of an acceptable scientific standard.

## References

[1] : Beanplot: A Boxplot Alternative for Visual Comparison of Distributions. *Journal of Statistical Software* .2008;28(Code Snippet 1) : 10.18637/jss.v028.c01 10.18637/jss.v028.c01

[2] : Data visualization in the neurosciences: overcoming the curse of dimensionality. *Neuron* .2012;74(4) : 10.1016/j.neuron.2012.05.001 603-8 10.1016/j.neuron.2012.05.001 22632718PMC4427844

# Reviewer report: Reviewer response for version 1

**DOI:** 10.21956/wellcomeopenres.16574.r35182

**Published:** 2019-04-15

**Authors:** Lisa M. DeBruine

**Affiliations:** 1 Institute of Neuroscience and Psychology, University of Glasgow, Glasgow, UK

This software tool article clearly describes a challenge (the prevalence of uninformative or misinformation plots) and presents a flexible solution with a strong logical rationale. Raincloud plots combine the advantages of box plots for inference, the advantages of distribution plots for assessing large-scale patterns (e.g., bimodality), and the advantages of scatter plots for assessing smaller-scale patterns (e.g., ordinality). A particular strength of this tutorial is the flexibility of the approach; several modular aspects are presented to make it easy to customise plots to fit the nature of the data, while keeping the presentation consistent enough that readers will be able to easily orient themselves to slightly different styles.

 I am only competent to assess the R tutorial, but it was easy to follow and very thorough. I look forward to seeing how this paper influences data visualisation in scientific research.

**Minor comments**

 I’m not sure I agree with the “data-to-ink ratio” argument against violin plots. An alternative perspective is not that they are mirrored density plots, but *centered* density plots. You don’t really save any ink with a density plot that takes up the same vertical and horizontal space as a violin plot; likewise, you can save equivalent ink by making the violin plot half it’s original width, rather than halving it down the middle. A better argument is that the density plot version makes the y-axis meaningful (although your raincloud plots omit the scale on the y-axis and I doubt many people use the actual density values).

**Code notes**

 I personally really hate code that installs anything (it’s a violation of my computer). I’d make the package setup chunk default to eval = FALSE and include a comment to turn it on if they want to install the missing packages (you can even run the first part and print a message if any packages are missing). But I guess there is a balance between making code easy for novices to run and best practices for respecting the user’s computer. I appreciate you taking the time to make sure installation only happens for uninstalled packages.

 In the code chunk “colour_rc” you add trim=FALSE to the flat violin, but don’t mention it and take it away in the next chunk.

 In the chunk “striated” you change the alpha of the violin and point between ap1 and ap2, but don’t explain why. Minimise irrelevant changes between steps to avoid confusing people and explain additions (the previous plot had no alpha) to avoid cargo-cult-like behaviour.

 Consider making the ggplot code less dense. I’ve put an example of what I mean below. Apart from that, the R tutorial is really clear and useful!

**Example of less dense (more readable) code:**

 p12 <- ggplot(rep_data, aes(x = group, y = score, fill = time))+

   geom_flat_violin(aes(fill = time),

                    position = position_nudge(x = .1, y = 0),

                    adjust = 1.5,

                    trim = FALSE,

                    alpha = .5,

                    colour = NA)+

   geom_point(aes(x = as.numeric(group)-.15, y = score, colour = time),

              position = position_jitter(width = .05),

              size = .25,

              shape = 20)+

   geom_boxplot(aes(x = group, y = score, fill = time),

                outlier.shape = NA,

                alpha = .5,

                width = .1,

                colour = "black")+

   geom_line(data = sumrepdat,

             aes(x = as.numeric(group)+.1,

                 y = score_mean,

                 group = time,

                 colour = time),

             linetype = 3)+

   geom_point(data = sumrepdat,

              aes(x = as.numeric(group)+.1,

                  y = score_mean,

                  group = time,

                  colour = time),

              shape = 18)+

   geom_errorbar(data = sumrepdat,

                 aes(x = as.numeric(group)+.1,

                     y = score_mean,

                     group = time,

                     colour = time,

                     ymin = score_mean-se,

                     ymax = score_mean+se),

                 width = .05)+

   scale_colour_brewer(palette = "Dark2")+

   scale_fill_brewer(palette = "Dark2")+

   ggtitle("Figure 12: Repeated Measures - Factorial (Extended)")+

   coord_flip()

Are the conclusions about the tool and its performance adequately supported by the findings presented in the article?

Yes

Is the rationale for developing the new software tool clearly explained?

Yes

Is the description of the software tool technically sound?

Yes

Are sufficient details of the code, methods and analysis (if applicable) provided to allow replication of the software development and its use by others?

Yes

Is sufficient information provided to allow interpretation of the expected output datasets and any results generated using the tool?

Yes

Reviewer Expertise:

I have substantial experience developing teaching materials for R stats, including ggplot.

I confirm that I have read this submission and believe that I have an appropriate level of expertise to confirm that it is of an acceptable scientific standard.
